# Biochemical characterization and identification of ferulenol and embelin as potent inhibitors of malate:quinone oxidoreductase from *Campylobacter jejuni*

**DOI:** 10.3389/fmolb.2023.1095026

**Published:** 2023-01-26

**Authors:** Augustin Tshibaka Kabongo, Rajib Acharjee, Takaya Sakura, Gloria Mavinga Bundutidi, Endah Dwi Hartuti, Cadi Davies, Ozan Gundogdu, Kiyoshi Kita, Tomoo Shiba, Daniel Ken Inaoka

**Affiliations:** ^1^ School of Tropical Medicine and Global Health, Nagasaki University, Nagasaki, Japan; ^2^ Department of Molecular Infection Dynamics, Institute of Tropical Medicine (NEKKEN), Nagasaki University, Nagasaki, Japan; ^3^ Department of Internal Medicine, Faculty of Medicine, Pharmacy and Public Health, University of Mbujimayi, Kinshasa, Congo; ^4^ Department of Parasitology, Institute of Tropical Medicine (NEKKEN), Nagasaki University, Nagasaki, Japan; ^5^ Program for Nurturing Global Leaders in Tropical and Emerging Communicable Disease, Graduate School of Biomedical Sciences, Nagasaki University, Nagasaki, Japan; ^6^ Department of Zoology, University of Chittagong, Chittagong, Bangladesh; ^7^ Department of Pediatrics, Kinshasa University Hospital, University of Kinshasa, Kinshasa, Congo; ^8^ Research Center for Genetic Engineering, National Research and Innovation Agency, West Java, Indonesia; ^9^ Faculty of Infectious and Tropical Diseases, London School of Hygiene and Tropical Medicine, London, United Kingdom; ^10^ Department of Host-Defense Biochemistry, Institute of Tropical Medicine (NEKKEN), Nagasaki University, Nagasaki, Japan; ^11^ Department of Biomedical Chemistry, Graduate School of Medicine, The University of Tokyo, Tokyo, Japan; ^12^ Department of Applied Biology, Graduate School of Science and Technology, Kyoto Institute of Technology, Kyoto, Japan

**Keywords:** malate:quinone oxidoreductase (MQO), drug target, enzyme inhibitor, membrane protein, biochemical characterization

## Abstract

*Campylobacter jejuni* infection poses a serious global threat to public health. The increasing incidence and antibiotic resistance of this bacterial infection have necessitated the adoption of various strategies to curb this trend, primarily through developing new drugs with new mechanisms of action. The enzyme malate:quinone oxidoreductase (MQO) has been shown to be essential for the survival of several bacteria and parasites. MQO is a peripheral membrane protein that catalyses the oxidation of malate to oxaloacetate, a crucial step in the tricarboxylic acid cycle. In addition, MQO is involved in the reduction of the quinone pool in the electron transport chain and thus contributes to cellular bioenergetics. The enzyme is an attractive drug target as it is not conserved in mammals. As a preliminary step in assessing the potential application of MQO from *C. jejuni* (CjMQO) as a new drug target, we purified active recombinant CjMQO and conducted, for the first time, biochemical analyses of MQO from a pathogenic bacterium. Our study showed that ferulenol, a submicromolar mitochondrial MQO inhibitor, and embelin are nanomolar inhibitors of CjMQO. We showed that both inhibitors are mixed-type inhibitors *versus* malate and noncompetitive *versus* quinone, suggesting the existence of a third binding site to accommodate these inhibitors; indeed, such a trait appears to be conserved between mitochondrial and bacterial MQOs. Interestingly, ferulenol and embelin also inhibit the *in vitro* growth of *C. jejuni*, supporting the hypothesis that MQO is essential for *C. jejuni* survival and is therefore an important drug target.

## 1 Introduction

*Campylobacter jejuni* is the most common cause of bacterial food-borne diseases worldwide, especially in developed countries, according to Centers for Disease Control and Prevention (CDC) and World Health Organization (WHO) (CDC, 2019; WHO, 2019) This gram-negative rod bacterium colonizes the intestinal tract of numerous animals, especially poultry such as chickens, which are the primary reservoir and source of human contamination (Kaakoush et al., 2015; Gao et al., 2017; Burnham and Hendrixson, 2018). Hence, improper handling or consumption of undercooked or raw poultry meat are the most prominent risk factors for *C. jejuni* infection in humans (Kaakoush et al., 2015; Gao et al., 2017). *C. jejuni* infection typically causes nonfatal diarrheal disease, but infection can lead to severe complications such as bacteraemia, reactive arthritis, irritable bowel disease, and Guillain-Barré syndrome with severe consequences (Kaakoush et al., 2015; Hlashwayo et al., 2021). Consequently, campylobacteriosis has a very high disease burden in humans (Sheppard and Maiden, 2015), and *C. jejuni* is a public health threat across the world. Recent studies have reported that *C. jejuni* is becoming increasingly resistant to antibiotics, such as fluoroquinolones, which are commonly used to treat campylobacteriosis (WHO, 2013; Facciolà et al., 2017; Shen et al., 2018). This new trend is partially attributable to the widespread use of these drugs to treat poultry and other livestock (WHO, 2013). Thus, in addition to general measures, such as the rational use of antibiotics, tackling *C. jejuni* and its related diseases is dependent upon the identification of new drugs with novel mechanisms of action and fewer side effects (Lipowska et al., 2019).

For decades, the energy metabolism has been investigated as a potential drug target in pathogens such as *Mycobacterium tuberculosis* and apicomplexan parasites, primarily because of the critical role that the energy metabolism plays in pathogens’ survival. As a *proof-of-concept*, some drugs targeting the oxidative phosphorylation and electron transport chain (ETC), such as bedaquiline and atovaquone, respectively, have already been approved (Mahajan, 2013; Nixon et al., 2013). The *C. jejuni* ETC is complex and highly branched, with a wide range of electron donors and alternative electron acceptors in addition to oxygen (Facciolà et al., 2017; Taylor and Kelly, 2019). Several of the dehydrogenases in the ETC are highly conserved, including flavodoxin-ubiquinone oxidoreductase (Nuo), sulphite oxidase, dihydroorotate dehydrogenase (DHODH), and malate:quinone oxidoreductase (MQO) (Liu et al., 2012; Gao et al., 2017; Burnham and Hendrixson, 2018). Among these, increasing attention has been paid to MQO, which plays roles in both the ETC and the tricarboxylic acid (TCA) cycle; specifically, it catalyses the oxidation of malate to oxaloacetate and reduces the menaquinone (MK) pool in the ETC, thereby contributing to cellular bioenergetics (van der Stel et al., 2017; Acharjee et al., 2021; Harold et al., 2022). Interestingly, since MQO is not conserved in mammals, in which malate dehydrogenase (MDH) is the only enzyme that is responsible for the oxidation of malate in the TCA cycle, MQO is an attractive drug target (Hartuti et al., 2018). Although controversial, MQO has been reported to be essential for the growth of bacteria such as *Pseudomonas aeruginosa* (Kretzschmar et al., 2002) and *Corynebacterium glutamicum* (Molenaar et al., 1998; Kabashima et al., 2013), and also in the survival of asexual stage of apicomplexan parasite, such as *Plasmodium falciparum* (Kretzschmar et al., 2002; Ke et al., 2015; Rajaram et al., 2022). MQO therefore appears to be well suited for use as a potential drug target (Samant et al., 2008; Acharjee et al., 2021). In addition, previous studies have reported that the TCA cycle is functional in *C. jejuni* (Liu et al., 2012), and the gene encoding the putative MQO gene in the *C. jejuni* genome has been identified (Pearson et al., 2007). Despite the availability of this information, no studies have been undertaken to either characterize this enzyme or evaluate its potential as a novel drug target for combatting campylobacteriosis.

In this study, we investigated the potential of MQO from *C. jejuni* (CjMQO) for use as a drug target. Specifically, we optimized the overexpression and purification of CjMQO, conducted biochemical characterization analyses, and investigated the inhibition mechanism of two CjMQO inhibitors. Our findings showed that CjMQO is potentially essential for the survival of *C. jejuni* and that it appears to be well suited for use as a drug target.

## 2 Materials and methods

### 2.1 Acquisition of the codon-optimized gene

The putative *C. jejuni* MQO (Cj0393c) sequence was obtained from the National Center for Biotechnology Information (NCBI). The amino acid sequence of CjMQO containing His10-SUMO and C-His10 at the N and C termini, respectively, was codon-optimized for expression in *Escherichia coli*. Three plasmid constructs were designed to contain the 1) His10-SUMO and 2) His6 tags on the N terminus, and 3) His10 tag on the C terminus. The primers were ordered from Hokkaido System Science Co., Ltd. (Sapporo, Japan) and the codon-optimized gene was synthesized and delivered in pUC-GW-Kan/His10SUMO-CjMQO-His10 (Azenta Life Sciences, South Plainfield, NJ, United States). As recommended by the manufacturers, all of the primers and CjMQO gene constructs were diluted with purified water to a final concentration of 50 μM and 50 ng/μL, respectively, and stored at −30°C until use.

### 2.2 Preparation of the plasmids

In order to prepare the three plasmid constructs, the CjMQO gene was amplified by PCR with Q5 High-Fidelity DNA polymerase using pUC-GW-Kan/His10SUMO-CjMQO-His10 as the template. For each construct, the PCR reaction mixture contained 1× Q5 reaction buffer and final concentrations of 0.25 mM dNTP mix, 0.2 ng/μL gene template, 0.04 U/µL Q5 High-Fidelity DNA Polymerase (New England BioLabs, Ipswich, MA, United States), 0.5 μM forward primer [P1 for the His10SUMO-CjMQO construct and P2 for both the CjMQO-His10 and His6-CjMQO constructs (Supplementary Figure S1)], 0.5 μM reverse primer [P3 for the CjMQO-His10 construct and P4 for both the His10SUMO-CjMQO and His6-CjMQO constructs (Supplementary Figure S1)], and water in a total volume of 50 µL. The PCR consisted of 98°C for 2 min, followed by 40 cycles of 95°C for 30 s, 53°C for 30 s, and 72°C for 1 min. Finally, the reaction was kept at 72°C for 10 min. The PCR products were separated by 1% (*w/v*) agarose gel electrophoresis and purified using the purification kit (Toyobo Co., Ltd., Osaka, Japan). Another gene construct, ‘tag-free’ (tag-free CjMQO), was amplified by the PCR. The His10SUMO-CjMQO and CjMQO-His10 gene constructs were cloned into plasmid pET101, while the tag-free CjMQO construct was cloned into pET151; the latter vector provides the His6 tag at the N-terminus. Finally, pET101/His10SUMO-CjMQO, pET101/CjMQO-His10 and pET151/His6-CjMQO were used separately to transform One Shot™ TOP10 Chemically Competent *E. coli* cells (Invitrogen™, Thermo Fisher Scientific, MA, United States) by the heat-shock method according to manufacturer’s instructions.

The *E. coli* that were successfully transformed with each plasmid were selected by plating on a Luria-Bertani agar (LB) plate using a carbenicillin marker and incubating overnight at 37°C. Ten colonies from each gene construct were picked, and confirmed by colony PCR (Quick Taq HS Dye Mix, Toyobo Co., Ltd.) using P6 forward primer (Supplementary Figure S1) and T7 reverse primer (Invitrogen™, Thermo Fisher Scientific, MA, United States) for all three gene constructs, following the manufacturer’s instructions. The colony PCR consisted of 95°C for 5 min, followed by 40 cycles of 95°C for 30 s, 57°C for 30 s, and 72°C for 2 min; then, the mixture was kept at 72°C for 10 min. Next, the identified positive colonies were further cultured separately in 5 mL LB broth supplemented with carbenicillin (100 μg/mL) at 200 rpm and 37°C for 15 h. Plasmids were extracted using a MagExtractor Plasmid purification kit (Toyobo Co., Ltd.) and sequenced (Fasmac Co., Ltd., Atsugi, Japan). The plasmid harbouring the correct CjMQO sequence was then used to transform BL21 Star™ (DE3) (Invitrogen™, Thermo Fisher Scientific) and NiCo21(DE3) (New England Biolabs) expression systems.

### 2.3 Transformation of expression hosts

The transformation of BL21 Star™ (DE3) and NiCo21(DE3) chemically competent *E. coli* cells was performed following the manufacturers’ protocols with few modifications. Briefly, a tube containing 50 µL of BL21 Star™ (DE3) or NiCo21(DE3) chemically competent *E. coli* cells was thawed on ice for 10 min. Then, 1 µL (30 ng) of each plasmid harbouring the CjMQO gene construct was added to the cell suspension and transformed by heat-shock method. Next, pre-warmed SOC medium (Invitrogen™, Thermo Fisher Scientific) was added to the mixtures to final volumes of 300 and 950 µL for the BL21 Star™ (DE3) and NiCo21(DE3) cells, respectively. The tubes were then incubated at 37°C for 60 min with shaking at 200 rpm. Finally, 250–300 µL of the cell mixture was spread onto a carbenicillin LB plate and incubated overnight at 37°C.

### 2.4 Optimization of recombinant CjMQO expression

First, to carry out the preculture, we picked one or two colonies from a carbenicillin plate to inoculate 150 mL of terrific broth (TB) medium [(12 g/L tryptone, 24 g/L yeast extract, 9.4 g/L K_2_HPO_4_, 2.2 g/L KH_2_PO_4_ supplemented with 5 mL/L of 80% (*v/v*) glycerol], and incubated for 15 h at 37°C with shaking at 200 rpm. Next, the preculture was inoculated into 3.6 L of the TB medium to an initial optical density of 0.1 at 600 nm (OD_600_). This culture was maintained under the same conditions as the preculture until the OD_600_ reached 0.6 to 0.8. Next, CjMQO expression was induced by adding isopropyl-β-D-thiogalactopyranoside (IPTG, Sigma-Aldrich Inc., Saint Louis, MO, United States) to a final concentration of 10 μM. The culture temperature was then reduced to 20°C and incubated for 40 h with shaking at 200 rpm.

### 2.5 Preparation of crude membrane fractions

All of the following steps were conducted at 4°C. When the OD_600_ reached 12 to 15, the cells were collected by centrifugation at 5,000 ×*g* (Hitachi Koki Co., Ltd., Shinagawa, Japan) for 15 min. Next, the supernatant was discarded and the pellet was suspended in lysis buffer [50 mM N-2-hydroxyethylpiperazine-N′-2-ethanesulfonic acid (HEPES, Dojindo Laboratories Co., Ltd.; Kumamoto, Japan) buffer pH 8.0, 0.25 mM phenylmethylsulphonyl fluoride (PMSF, Sigma-Aldrich Inc.)] at a proportion of 18–20 mL per 5 g of wet bacterial pellet. Then, the bacterial cells were disrupted at 180 MPa using a pre-cooled French Press (Ohtake Works Ltd., Sakado, Japan), and centrifuged at 30,000 ×*g* for 30 min to remove unbroken cells and debris (Hitachi Koki Co., Ltd.). Next, the supernatant was subjected to ultracentrifugation at 200,000 ×*g* for 1 h (Hitachi Koki Co., Ltd.). After removing the supernatant (cytosol), the pellet (membrane fractions) was suspended with 6 mL of the resuspension buffer [50 mM HEPES pH 8.0, 5 mM imidazole (Wako Pure Chemical Industries, Ltd., Osaka, Japan), 150 mM KCl (Wako Pure Chemical Industries, Ltd.)] and homogenized (Ikemoto Rika Kyogo Co., Ltd., Japan). Next, the membrane fractions were transferred into a falcon tube and supplemented with ice-cold glycerol (Wako Pure Chemical Industries, Ltd.) and flavin adenine dinucleotide (FAD) [Tokyo Chemical Industry Co., Ltd. (TCI), Tokyo, Japan] to a final concentration of 50% (*v/v*) and 200 μM, respectively, and stored at −30°C until use.

### 2.6 Purification of recombinant CjMQO

The membrane fractions (15 mL, 20.1 mg/mL) were diluted with a 1.5 volume of the dilution buffer (50 mM HEPES pH 8.0, 200 μM FAD) and mixed with solubilizing buffer [50 mM HEPES pH 8.0, 2% (*w*/*v*) n-Octyl-β-D-glucoside (OG, Dojindo Laboratories Co., Ltd.), 10 mg/mL 1,2-diacyl-sn-glycero-3-phosphocholine (Sigma-Aldrich Inc), 200 μM FAD] at a ratio of 1:1. The solubilised membranes were gently mixed using the rotator (Rotator RT-50; Taitec Co., Koshigaya, Japan) for 30 min. After solubilization, the sample was ultracentrifuged at 200,000 ×*g* for 1 h to remove insoluble proteins and membrane debris, and the supernatant was transferred into a tube containing 2 mL of cOmplete His-Tag Purification Resin (Roche Diagnostics GmbH, Mannheim, Germany) pre-equilibrated with buffer A [50 mM HEPES pH 8.0, 0.1% (*w*/*v*) OG, 200 μM FAD, 10 mM imidazole)]. The CjMQO was allowed to bind to the resin by mixing gently (∼20–30 rpm) for 3 h and then poured into a 20 mL chromatography column (Poly-Prep, Bio-Rad Laboratories, United States). The unbound protein was collected as the flow through, and the resin was washed with 60 mL buffer A, then with 30 mL buffer B [50 mM HEPES pH 8.0, 0.1% (*w*/*v*) OG, 200 μM FAD, 40 mM imidazole]. The protein was eluted using 15 mL of buffer C (50 mM HEPES pH 8.0, 0.1% (*w*/*v*) OG, 200 μM FAD, 200 mM imidazole) and collected using 30 kDa molecular weight cut-off centrifugal filters (Amicon Ultra-15, Merck Millipore Ltd., Carrigtwohill, Ireland). The eluate was then concentrated by ultrafiltration until the volume was reduced to 200–300 μL. The concentrated eluate was collected and mixed with glycerol to a final concentration of 50% (*v*/*v*), the FAD was adjusted to a final concentration of 200 μM, and kept at −30°C until use. To obtain the purified protein for FAD quantification study, the FAD was removed from buffers A, B, and C, and purified as described above.

### 2.7 Protein quantification, FAD content determination, and electrophoresis of CjMQO

The protein was quantified by the Bradford assay (Bio-Rad Laboratories) using bovine serum albumin (BSA; Takara Inc. Shiga, Japan) as the standard according to the manufacturer’s instructions. The ratio of FAD per CjMQO was estimated spectroscopically using the protein purified in absence of FAD and the extinction coefficient of 11.1 mM^-1^ cm^-1^ at 450 nm (Supplementary Figure S2) (Takashima et al., 2002).

Purified CjMQO was analysed by sodium dodecyl sulphate 12% (*w/v*) polyacrylamide gel electrophoresis (SDS-PAGE) with fractions from all other purification steps. All of the samples, except the wash B and concentrated eluate, were prepared to a final protein concentration of 1 μg/μL using 1 × SDS-PAGE loading buffer [62.5 mM Tris-HCl pH 6.8, 10% (*v/v*) glycerol, 2.5% (*w*/*v*) SDS (Wako Pure Chemical Industries, Ltd.), 0.002% (*v*/*v*) bromophenol blue (BPB, Wako Pure Chemical Industries, Ltd.), and 0.71 M β-mercaptoethanol (Wako Pure Chemical Industries, Ltd.)]. After being heated for 10 min at 95°C, samples from each purification step were loaded onto SDS-PAGE gel together with the protein ladder (Precision Plus Protein™ All Blue Prestained Protein Standards, Bio-Rad Laboratories), and run at 25 mA constant. Then the protein was stained overnight with GelCode™ Blue Safe Protein stain (Thermo Fisher Scientific Inc.).

To determine the oligomeric state of CjMQO, high-resolution clear native electrophoresis (hrCNE) was performed by adapting the protocol reported for *Toxoplasma gondii* MQO (Acharjee et al., 2021). Briefly, the purified CjMQO was diluted to final concentrations of 0.3, 0.2, 0.1, and 0.05 μg/μL in a buffer containing 50 mM HEPES pH 8.0, 0.6% (*w/v*) OG, 5% (*v/v*) glycerol, 0.05% (*w/v*) ponceau S (MP Biomedicals LLC, France), and 0.05% (*w/v*) sodium deoxycholate (DOC; Nacalai Tesque Inc., Kyoto, Japan). Next, CjMQO was loaded on a 4%–16% (*w/v*) Bis-Tris gradient gel (NativePAGE™, Invitrogen™, Thermo Fisher Scientific) with 3, 2, 1, and 0.5 μg per lane, together with a protein standard (NativeMark™ Protein Standard, Invitrogen™, Thermo Fisher Scientific); duplicates of all samples were loaded [one for Coomassie Brilliant Blue (CBB) and another for MQO activity staining]. The gel was run at a constant current of 150 V at 200 W (WSE 3200, Atto Co.) in 1 × NativePAGE^TM^ running buffer (Life Technologies, Carlsbad, CA, United States) with (cathode) or without (anode) 0.05% (*w/v*) n-dodecyl-β-D-maltoside (DDM, Dojindo Laboratories Co., Ltd.) and 0.05% (*w/v*) DOC supplementations. The in-gel CjMQO activity staining was performed by washing the gel three times for 5 min each in 5 mM HEPES buffer pH 8.0, followed by incubation with 15 mL of 10 mM nitro-blue tetrazolium chloride (NBT, Wako Pure Chemical Industries, Ltd.) for 5 min. Finally, phenazine methyl sulphate (PMS, Tokyo Chemical Industry Co., Ltd.) and sodium malate (Wako Pure Chemical Industries, Ltd.) were added to final concentrations of 0.1 mg/mL and 10 mM, respectively. The gel container was then kept in a static position and in a dark place overnight at room temperature. The gel was washed with water and checked for visible bands the next day. Since CjMQO can directly reduce PMS in the absence of quinones, this method can be specific for detection of MQO, because a similar method for MDH activity staining requires the presence of NADH to mediate the PMS reduction (Kohsaka et al., 1992).

### 2.8 Optimization of CjMQO assay conditions

The linearity range of CjMQO activity was evaluated using a dose-response curve following a method reported previously with several modifications (Hartuti et al., 2018; Acharjee et al., 2021). The spectrophotometric assay was performed in triplicate at 37°C with a spectrophotometer (V760, Jasco Co., Tokyo, Japan) connected to an open bath circulator (Julabo ED, Julabo Labortechnik GmbH, Germany). The CjMQO concentrations ranged from 0.01 to 2 μg/mL in a 1 mL reaction mixture containing 50 mM HEPES (pH 7.0), 20 μM decylubiquinone (dUQ, Sigma-Aldrich Inc.), 1 mM potassium cyanide (KCN, Sigma-Aldrich Inc.) and 120 μM 2,6-dichlorophenolindophenol (DCIP, Sigma-Aldrich Inc.). The reaction was started by adding 10 mM sodium malate to the reaction mixture, and the reduction of DCIP was measured at 600 nm. The specific activity was calculated by using the extinction coefficient ( $\varepsilon_{600}$ ) of DCIP of 21 mM^-1^ cm^-1^. The optimum temperature for the activity of purified CjMQO was determined in triplicates under different temperatures as indicated above at fixed concentration of 0.2 μg/mL of purified CjMQO.

The optimum pH was determined as described previously (Hartuti et al., 2018; Acharjee et al., 2021) by measuring CjMQO activity at different pH values using 50 mM of HEPES-NaOH (pH 6.8–8.4), Tris-HCl (pH 6.9–9.0), sodium phosphate (NaPi, pH 5.8–8.0), potassium phosphate (KPi, pH 5.8–8.0), MOPS-NaOH (pH 6.5–8.9), and CHES-NaOH (pH 8.6–10.0) buffers at 37°C and 0.2 μg/mL of purified CjMQO with a multi-mode microplate reader (SpectraMax Paradigm, Molecular Devices, San Jose, CA, United States).

### 2.9 Determination of enzyme steady-state kinetic parameters and reaction mechanism

The steady-state kinetic parameters of CjMQO were determined for malate and ubiquinone (UQ) with different side chain length (UQ0, UQ1, UQ2, UQ4, and dUQ; Supplementary Figure S3). The reaction mixture contained UQ, 1% (*v/v*) ethanol, 50 mM MOPS, 1 mM KCN and 0.2 μg/mL of purified CjMQO at the optimal pH (pH 7.0) and physiological temperature (37°C). The reduction of quinones was measured through the decrease in the absorbance at 278 nm ( $\varepsilon_{278}=$ 15 mM^-1^ cm^-1^). The Michaelis constant (*K*_m_) and maximum velocity (*V*_max_) of CjMQO for UQs were determined by varying the concentrations of UQs from 0.1 to 100 μM with the malate concentration fixed at 10 mM.

Next, *K*_m_ and *V*_max_ of CjMQO for malate were determined by varying the concentrations from 0.5 to 50 mM at fixed concentrations of different UQs (100 μM for UQ0 and UQ1 and 10 μM for dUQ and UQ2). The apparent steady-state kinetic parameters were calculated by Michaelis-Menten equation using GraphPad Prism software (ver. 9.3.1, GraphPad Software, Inc., San Diego, California, United States). Following the protocol previously described for TgMQO (Acharjee et al., 2021), the reaction mechanism of CjMQO was analysed using malate and UQ1 as substrates. Briefly, CjMQO activity was measured at different UQ1 concentrations (2, 5, 10, 20, and 50 μM) with malate concentrations fixed at 0.5, 2, and 5 mM; the data were then analysed using a double reciprocal plot (Lineweaver-Burk plot) produced by utilizing intercept and slope calculated from steady-state kinetics parameters obtained by fitting the data to Michalis-Menten equation with GraphPad Prism software. The type of reaction mechanism was determined based on the characteristics of the lines plotted in the double reciprocal plot.

### 2.10 Ferulenol and embelin inhibit CjMQO and also the growth of *C. jejuni*

Half-maximal inhibitory concentrations (IC_50_) of ferulenol (AdipoGen Life Sciences, Inc., Epalinges, Switzerland) and embelin (Indofine Chem Co, Inc.; Hillsborough, NJ, United States) on CjMQO were determined by measuring the residual activity at 278 nm. The enzymatic activity of CjMQO was measured in the presence of different concentrations of ferulenol or embelin (0.01 nM–100 μM) and the reaction mixture, which contained 50 mM MOPS buffer pH 7.0, 1 mM KCN, 100 μM UQ1, and 0.2 μg/mL CjMQO. The reaction was started by the addition of 10 mM malate (final concentration). The data were fitted to a log(inhibitor) vs. response -- Variable slope model using a nonlinear regression method in GraphPad Prism software.

Frozen stock of *C. jejuni* strain ATCC29428 (equivalent to JCM 2013) in 10% (*v/v*) glycerol, 10% (*v/v*) foetal bovine serum (Nichirei Biosciences Inc., Tokyo, Japan), 80% (*v/v*) *Brucella* broth (Oxoid Ltd., United Kingdom) was thawed and cultured on a Columbia blood agar base (Oxoid Ltd., Hampshire, United Kingdom) enriched with Skirrow *Campylobacter*-selective supplement (Oxoid Ltd.) and 6% (*v/v*) lysed horse blood (Kanto Chemical Co., Inc., Tokyo, Japan). The Skirrow *Campylobacter*-selective supplement consisted of 5.0 mg vancomycin, 2.5 mg trimethoprim, and 1250 IU polymyxin B that was reconstituted in 500 mL nutrient medium. The blood agar plate was incubated at 37°C under microaerophilic conditions (5% O_2_, 5% CO_2_, 90% N_2_) for 48 h. Next, several colonies were transferred to 20 mL of preculture Mueller Hinton broth (Oxoid Ltd.) enriched with Skirrow *Campylobacter*-selective supplement. The antibiotic sensitivity of *C. jejuni* ATCC29428 was determined using a resazurin assay in a 96-well plate. Briefly, *C. jejuni* ATCC29428 was cultured in 125 μL of Mueller Hinton broth for 24 h, starting with an initial OD of 0.01 in the presence of different antibiotics (final concentrations of 0.001–100 μg/mL) in triplicates. Kanamycin and ciprofloxacin (Wako Pure Chemical Industries, Ltd.) were diluted in DMSO to keep the final concentration of DMSO in the culture at 1% (*v/v*). We used a medium without bacteria and antibiotics (only DMSO) to check the background (i.e., as a blank). The 96-well plate alongside one gas-generator sachet (AnaeroPack-Microaero, Mitsubishi Gas Chemical Co., Tokyo, Japan) was placed inside a container (Rectangle Jar, Standard, Mitsubishi Gas Chemical Co.) sealed completely to maintain microaerophilic conditions for 24 h at 37°C while shaking at 120 rpm. After 24 h, 25 μL of 0.1 mg/mL resazurin (Tokyo Chemical Industry Co., Ltd) was added to the culture and mixed well at 800 rpm for 1 min, the mixture was then incubated under microaerophilic conditions at 37°C for 30 min (Prescyto, Taitec, Saitama, Japan). Next, fluorescence was measured using a microplate reader (SpectraMax Paradigm, Molecular Devices) at 540 nm and 590 nm as excitation and emission wavelengths, respectively. The data were imported into GraphPad Prism software (GraphPad Software, Inc) and fitted to a log (inhibitor) vs. response -- Variable slope model to calculate the IC_50_ of the antibiotics using a nonlinear regression method. Similarly, *C. jejuni* ATCC29428 was grown in the presence of varying concentrations of ferulenol and embelin; the residual growth and IC_50_ were determined under the same conditions as described above for antibiotics. Kanamycin and ciprofloxacin were mixed to a concentration of 50 μg/mL each and used as the positive control (100% inhibition).

The inhibition mechanism of the two compounds were investigated as described in a previous study (Acharjee et al., 2021). First, the inhibition mechanism versus UQ1 was assayed separately in a reaction mixture containing 50 mM MOPS pH 7.0, 1 mM KCN and 0.2 μg/mL of purified CjMQO containing a range of different ferulenol (0, 0.01, 0.02, and 0.04 μM) and embelin (0, 0.05, 0.1, and 0.15 μM) concentrations. Enzyme activity was measured at varying concentrations of UQ1 (5, 10, 20, 50, and 100 μM) and the reaction was initiated with 10 mM malate. Second, the inhibition mechanism versus malate was determined at 50 μM of UQ1, and the reaction was initiated with varying concentrations of malate (5, 10, 15, and 20 mM) and monitored at 278 nm, in singlets. The steady-state kinetics parameters, in the presence of inhibitors, were used to produce the Lineweaver-Burk plot. Next, the data were fitted to a mixed-type inhibition equation by a nonlinear-regression method using GraphPad Prism software. The intercept and slope from the primary plot (Lineweaver-Burk plot), were replotted as a function of the inhibitor. Finally, additional analysis was performed based on Dixon and Cornish Bowden plots in addition to the plot of Lineweaver-Burk to differentiate the types of inhibition mechanisms.

## 3 Results and discussion

### 3.1 Purified *C. jejuni* MQO exhibits high specific activity

MQO is an attractive drug target as it is essential for the survival of numerous pathogens and is not conserved in mammalian cells. Our results provide the first biochemical study of recombinant MQO from *C. jejuni*, which was overexpressed in the membrane fraction of NiCo21(DE3) *E. coli* and purified to homogeneity after optimization.

As a first step, we optimized the position of His10 or His6 tags to improve the expression and activity of CjMQO. Although all the constructs increased the MQO activity in membrane fractions from the BL21 Star™ (DE3) cells, the specific activity was found to be highest when CjMQO was expressed with a His6-tag at the N-terminus (pET151/H6-CjMQO) (Supplementary Figure S4). Next, we determined that 10 μM IPTG was the optimal concentration for the induction of CjMQO expression as it resulted in the highest specific activity (Supplementary Figure S5A). As claimed by the manufacturer, the NiCo21(DE3) strain yields His-tagged recombinant proteins with fewer common contaminants after affinity purification. In the present study, the membrane fractions of NiCo21(DE3) cells showed higher specific activity when compared to BL21Star™ (DE3) cells (Supplementary Figure S5B); hence NiCo21(DE3) was selected as the expression host used for purification of CjMQO. In *E. coli*, both endogenous MQO and MDH are simultaneously expressed (van der Rest et al., 2000). Therefore, when the expression conditions were set, the specific activity of the recombinant CjMQO in the membranes (22 ± 0.3 μmol/min/mg) was 737-times higher than that of the endogenous MQO (0.03 ± 0.01 μmol/min/mg) detected in membrane fractions of *E. coli* that harboured the empty plasmid (pET151) (Table 1), demonstrating the effectiveness of the expression system used in this study.

**TABLE 1:** Purification table of CjMQO.

Fraction	Total protein (mg)	Total activity (μmol/min/mg)	Specific activity (μmol/min/mg)	Yield^1^ (%) (Lysate)	Yield^2^ (%) (Membrane)	Purification^3^ (x-fold)
Lysate	1,820	12,750	7.0 ± 0.6	100	−	1.0
Clarified lysate	1,350	10,040	7.5 ± 0.2	79	−	1.1
Cytosol	800	940	1.2 ± 0.2	7.4	−	0.2
Membrane	297	6,570	22 ± 0.3	52	100	3.2
Flow through	279	3,510	13 ± 0.5	28	53	1.8
Wash A	16	138	8.7 ± 0.4	1.1	2.1	1.2
Wash B	1.2	13.4	11 ± 0.7	0.1	0.2	1.6
Concentrated eluate	2.0	250	125 ± 5.4	2.0	3.8	18
Control membrane*	21	0.6	0.03 ± 0.01	NA	NA	NA

The specific activity of CjMQO was determined spectrophotometrically in the presence of 50 mM HEPES buffer pH 7.0 at 37°C using a DCIP reduction assay (600 nm). The values reported in this table are the average of three measurements. The yield and purification x-fold are calculated from the total activity in the crude lysate^1^, the membranes^2^ and the specific activity of the lysate^3^. Control membranes* are membrane fractions of NiCo21(DE3) carrying the pET151 empty vector cultured under the same conditions as NiCo21(DE3)-pET151/His6-CjMQO. NA: not applicable.

**FIGURE 1:**
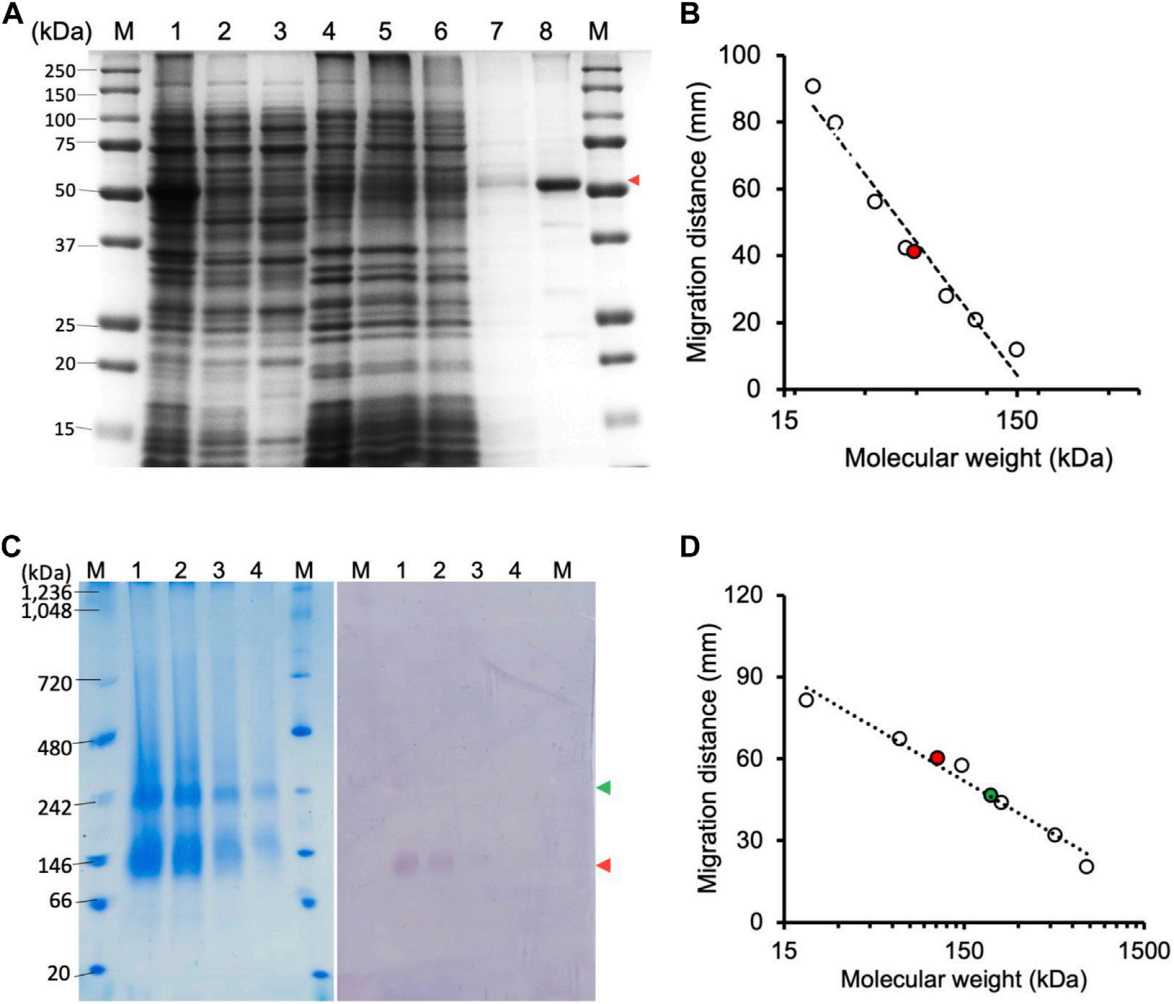
Electrophoresis of CjMQO. **(A)** SDS-PAGE analysis of different steps of CjMQO purification (expected size 54 kDa). Lane M, marker (6 μg); Lane 1, Lysate (10 μg); Lane 2, Clarified lysate (10 μg); Lane 3, Cytosol (10 μg); Lane 4, Membrane fractions (10 μg); Lane 5, Flow through (10 μg); Lane 6, Concentrated Wash A (10 μg); Lane 7, Concentrated Wash B (0.1 μg); and Lane 8, Concentrated eluate (1 μg). The red arrow indicates the band corresponding to CjMQO. **(B)** Semi-logarithmic plot of migration distance used to calculate the molecular weight of CjMQO under denaturation conditions. **(C)** Coomassie brilliant blue staining on a high-resolution clear native electrophoresis (hrCNE) gel of purified CjMQO (left) and in-gel activity staining of purified CjMQO (right). Lane M, Marker (5 μg) and purified CjMQO: Lane 1 (3 μg); Lane 2 (2 μg); Lane 3 (1 μg); and Lane 4 (0.5 μg). The red and green arrows indicate the location corresponding to the dimer and tetramer of CjMQO, respectively. **(D)** Semi-logarithmic plot of migration distance for calculating the molecular weight of CjMQOs under native conditions.

The CjMQO was solubilized and purified in presence of OG detergent during all the purification steps, as other commonly used detergents such as DDM, Triton X-100, and CHAPS resulted in complete loss of the enzymatic activity after solubilization from the membranes. Judging from the SDS-PAGE results, the purity of CjMQO in the elution was approximately 95% (Figure 1A). The estimated molecular weight was 54.3 kDa (Figure 1B), which is consistent with the size calculated from the amino acid sequence. After optimizing the purification conditions, 2 mg of highly active CjMQO (125 ± 5.4 μmol/min/mg) was purified from a 3.6 L of TB culture (∼41.4 g of wet bacterial pellet) (Table 1). The purified CjMQO had an FAD content to protein ratio ranging between 0.93 and 1.16 for the purified enzyme displaying specific activity ranging from 104 to 119 μmol/min/mg, indicating one molecule of CjMQO binds one FAD. Other preparations where the specific activity was decreased to 80 μmol/min/mg, the CjMQO:FAD ratio also decreased to 0.7. In such case, incubation of CjMQO with FAD did not restore the activity, indicating that the release of FAD from the enzyme is an irreversible process. Moreover, the CjMQO purified in this study showed one of the highest activities reported in MQOs to date after that observed in *Acetobacter* sp. SKU 14 (993 μmol/min/mg) (Shinagawa et al., 2002). In a DCIP reduction assay, CjMQO had an activity of 125 μmol/min/mg (Table 1), which is very high compared to MQO purified from *C. glutamicum* (9.60 μmol/min/mg) (Molenaar et al., 2000), *Bacillus* spp. DSM 465 (0.120 μmol/min/mg) (Ohshima and Tanaka, 1993), *Bacillus* sp. PS3 (24.6 μmol/min/mg) (Kabashima et al., 2013), and *T. gondii* (22.0 μmol/min/mg) (Acharjee et al., 2021).

It is generally accepted that *C. jejuni* does not utilize glucose as carbon source and rely on an active gluconeogenesis from oxaloacetate for the production of glucose-6-phosphate, which is used to feed the pentose phosphate pathway and polysaccharide biosynthesis (Velayudhan and Kelly, 2002). Under microaerophilic environment, *C. jejuni* uses aspartate as the primary carbon source, which is converted to fumarate used 1) for fumarate respiration by the two types of fumarate reductases (FrdABC or MfrABE) or 2) by fumarase and MQO to produce oxaloacetate required for gluconeogenesis (Guccione et al., 2017). In this condition, MQO could have an additional advantage to contribute for the generation of proton motive force when coupled with FrdABC, as the malate oxidation and fumarate reduction occur on the cytoplasmic side whilst menaquinol oxidation occurs on the periplasmic side (distal haem of FrdC subunit), similarly to the succinate/nitrate oxidoreductase system from *Paracoccus denitrificans* (Simon et al., 2008). Interestingly, MDH which is conserved in *C. jejuni* and thermodynamically favours the conversion of oxaloacetate to malate, has been reported to be downregulated under microaerophilic environment (Guccione et al., 2017). Therefore, the high specific activity of CjMQO found in this study would support proper flux of carbons to gluconeogenesis, and shuttling electrons to the ETC.

### 3.2 Determination of *C. jejuni* MQO oligomeric state

The high-resolution clear native electrophoresis (hrCNE) analysis revealed two distinctive bands after CBB staining (Figure 1C, left) and in-gel CjMQO activity (Figure 1C, right) staining. The lower band corresponded to a dimer (107.5 kDa) and the upper band to a tetramer (212 kDa) of CjMQO, which was inferred based on the migration distance (Figure 1D). The oligomeric state of proteins plays a critical role in their function. In living cells, proteins can exist as oligomers that consist of subunits that are either different (hetero-oligomer) or similar (homo-oligomer) to each other. Homo-oligomers are widespread in nature (Danielli et al., 2020). MQOs purified from other bacterial species have been shown to have different numbers of subunits in their oligomeric state, such as homodimers (*Acetobacter* sp. SKU 14 and *Bacillus* sp. PS3), tetramers and dimers of tetramers (*T. gondii*) and decamers (*Bacillus* sp. DSM 465) (Ohshima and Tanaka, 1993; Shinagawa et al., 2002; Kabashima et al., 2013; Acharjee et al., 2021). In this study, the findings of activity staining on hrCNE showed that the dimer of CjMQO appeared to be more active than the tetramer state. This observation suggests that the dimeric form could be more stable than the tetrameric form and might constitute the functional unit of CjMQO when the binding sites of quinone (on the hydrophobic face) and malate (on the hydrophilic face) are solvent-accessible. Another plausible explanation for the higher activity of the dimeric than tetrameric forms is that the active site of this enzyme could be located at the dimerization interface, and the binding site of one or both substrates might be hidden by tetramerization at the dimer-dimer interface, preventing the substrate(s) from binding to the enzyme and likely explaining the low activity of the tetramer observed in this study. Therefore, determination of CjMQO crystal structure can provide further insights into the relationship between oligomeric state and MQO activity.

### 3.3 Optimization of *C. jejuni* MQO activity assay conditions

**FIGURE 2:**
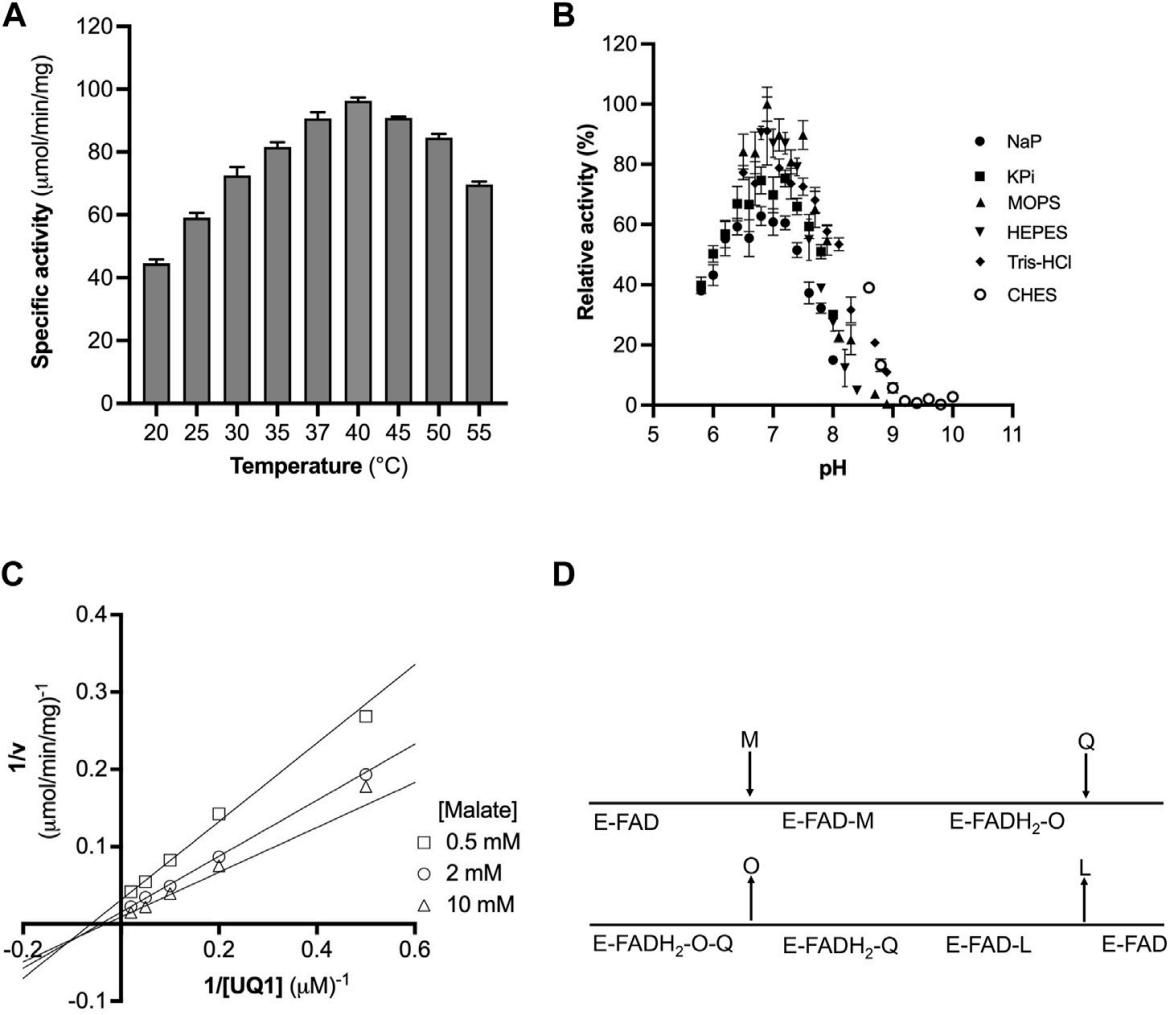
Optimization of CjMQO temperature and pH. **(A)** The optimal temperature of CjMQO was assayed spectrophotometrically. The enzymatic activity at each point was determined at varying temperatures and in triplicate. **(B)** The optimal buffer and pH conditions were determined using different buffers and pH values at 37°C; values represent the average of triplicate measurements. **(C)** Analysis of the kinetic mechanism of CjMQO using a double reciprocal plot. The activity of purified CjMQO was measured by varying UQ1 concentrations at fixed malate concentrations (i.e., 0.5, 2, and 10 mM). The straight lines intersecting below the X-axis in the third quadrant indicate a bisubstrate sequential Bi-Bi reaction mechanism. **(D)** Schematic representation of the Bi-Bi reaction mechanism in which malate (M) and quinone (Q) are the substrates of the enzyme (E) and oxaloacetate (O) and quinol (L) are the products. E-FAD and E-FADH_2_ represent the enzyme with bound FAD in its oxidized and reduced form, respectively.

The dependency of the initial velocity on the concentration of purified CjMQO in the assay was evaluated and revealed a linear response between 0.01 and 0.2 μg/mL with an *R* ^2^ of 0.999, while the specific activity remained stable up to 1 μg/mL (Supplementary Figure S6). At high concentrations, we observed a decrease in the specific activity and the linearity of the response. Thus, the concentration of purified CjMQO was fixed at 0.2 μg/mL for further steps in this study. The optimal temperature for the activity of the purified CjMQO was 40°C; however, the enzyme retained 60% and 93% of its maximal activity at ambient (25°C) and human body (37°C) temperatures, respectively (Figure 2A). This optimal temperature was lower than values published for MQOs from *T. gondii* (50°C), thermophilic *Bacillus* sp. DSM 465 (55°C), and *Bacillus* sp. PS3 MQO (45°C) (Ohshima and Tanaka, 1993; Kabashima et al., 2013; Acharjee et al., 2021). It was superior to the temperature reported for *P. taetrolens* MQO (30°C) and *P. falciparum* MQO (37°C) (Hartuti et al., 2018; Oh et al., 2020). *C. jejuni* resides in the intestinal tract of chickens and birds, which have a body temperature of 42°C (Burnham and Hendrixson, 2018); consequently, the optimal temperature of CjMQO (40°C) is likely to reflect the lifestyle of this bacterium in avian hosts. The optimal pH for the CjMQO activity was investigated and showed a distinct bell-shaped response curve, with a maximum activity at pH 7.0 for most of the buffers tested, though MOPS showed the highest activity (Figure 2B). This pH is slightly lower than, albeit similar to, most optimal pH values reported for MQO from other bacteria and parasites. For example, the optimum pH was 7.5 for *Bacillus* sp. PS3 MQO and in the range of 7.0–8.0 for *Pseudomonas taetrolens*, *P. falciparum* and *T. gondii* MQOs (Kabashima et al., 2013; Hartuti et al., 2018; Oh et al., 2020; Acharjee et al., 2021).

In the present study, DCIP was reduced by CjMQO in the absence of quinones, yielding a specific activity that corresponded to approximately one-third of the activity recorded in the presence of both DCIP and dUQ (Supplementary Figure S7). Hence, we decided to use a direct quinone reduction assay for the subsequent steps in this study. As hypothesized by Acharjee et al., this observation could be explained by DCIP being able to accept electrons directly at the quinone binding site on MQO (Acharjee et al., 2021).

### 3.4 Apparent steady-state kinetic parameters of the purified *C. jejuni* MQO

The affinity of CjMQO for UQ0, UQ1, UQ2, UQ4, and dUQ was estimated through Michaelis constant (*K*_m_) values which were 168, 46, 4.6, 0.8, and 8.5 μM, while the maximal velocity was 49, 107, 108, 10, and 76 μmol/min/mg, respectively (Table 2; Supplementary Figures S8A–C). This affinity of CjMQO for ubiquinones indicated that the longer the side chain of benzoquinone is, the smaller the *K*_m_ value is, meaning that CjMQO had a higher affinity for quinones with long side chains. The affinity of CjMQO for different quinones was stronger than that reported for TgMQO (with *K*_m_ values of 168 *vs.* 225 μM for UQ0, 46 *vs.* 116 μM for UQ1, 8.5 *vs.* 17 μM for dUQ). In addition, the maximal velocity (*V*_max_) of CjMQO for ubiquinones was higher than that of TgMQO (49 *vs.* 13 μmol/min/mg for UQ0, 107 *vs.* 44 μmol/min/mg for UQ1, 76 *vs* 12 μmol/min/mg for dUQ) (Acharjee et al., 2021). Furthermore, in the presence of UQ0, UQ1, UQ2, and dUQ, the *K*_m_ of CjMQO for malate was 298, 882, 1,031, and 1,209 μM, respectively (Table 2; Supplementary Figures S9A, B). These values indicate that the affinity of CjMQO for malate is weaker than that from TgMQO, but slightly stronger than PfMQO. Indeed, the *K*_m_ values of CjMQO for malate substrate were two to three-times higher in CjMQO than in TgMQO (i.e., 882 *vs.* 370 μM; 1,031 *vs.* 637 μM; 1,209 *vs.* 466 μM in the presence of UQ1, UQ2, and dUQ, respectively) (Acharjee et al., 2021) and five-times lower than that of PfMQO (1,209 *vs.* 5,990 μM for dUQ) (Hartuti et al., 2018). Moreover, substrate inhibition was observed above 10 μM for UQ2 and dUQ and 2 μM for UQ4 (Supplementary Figure S8D) possibly due to the low solubility in the assay condition. Consistently, when UQ2 was used as the electron acceptor highest catalytic efficiency (*K*_cat_/*K*_m_) of 107 s^-1^ μM^-1^ was observed, which decreased to 53 s^-1^ μM^-1^ when UQ4 was used (Table 2).

**TABLE 2:** Summary of apparent steady-state kinetic parameters of CjMQO.

Substrates		*K*_m_	*V*_max_	*k*_cat_	*k*_cat_ */K*_m_
Fixed	Variable	(μM)	(μmol/min/mg)	(sec^−1^)	(sec^−1^.μM^−1^)
Malate	UQ0	168 ± 12.0	49.0 ± 2.4	219 ± 13.1	1.30
Malate	UQ1	46.0 ± 3.1	107 ± 5.2	480 ± 18.7	10.5
Malate	UQ2	4.60 ± 1.0	108 ± 10	485 ± 42.0	107
Malate	UQ4	0.80 ± 0.2	10.0 ± 1.0	43.9 ± 11.4	53.0
Malate	dUQ	8.50 ± 2.0	76.0 ± 9.0	343 ± 40.4	40.2
UQ0	Malate	298 ± 18	15.0 ± 0.2	67.0 ± 1.10	0.20
UQ1	Malate	882 ± 49	112 ± 1.3	502 ± 55.6	0.60
UQ2	Malate	1,031 ± 93	86.0 ± 1.7	389 ± 7.60	0.40
dUQ	Malate	1,209 ± 160	47.0 ± 1.4	210 ± 19.2	0.20

*K*_m_, *k*_cat_ and *V*_max_ values represent the average value of reactions performed in triplicate. Data were fitted to Michaelis-Menten equation from GraphPad Prism 9.0. Substrate inhibition was observed over 10 μM for UQ2 and dUQ, and 2 μM for UQ4 (Supplementary Figure 8D). Therefore, the *K*_m_ and *V*_max_ shown in the table are obtained from the data set which substrate inhibition is not observed (Supplementary Figure 8C). SD: standard deviation.

In this study, we were unable to test the activity of CjMQO towards the menaquinone found naturally in *C. jejuni*, because of its highly reactive nature with oxygen (once reduced). In addition, such assay would require a sophisticated experimental apparatus where the enzyme activity with menaquinone is assayed under entirely anaerobic conditions (Wissenbach et al., 1990), which was not available in the lab. Typically, ubiquinone-dependent enzymes, such as human dihydroorotate dehydrogenase (Peres et al., 2017) and *bc*_1_ complexes from *P. falciparum* and *Echinococcus multilocularis* (Goodman et al., 2016; Enkai et al., 2021) are inhibited by naphthoquinones. Interestingly, the ubiquinone-dependent mitochondrial MQOs from apicomplexan parasites have a high amino acid identity with MQO from *C. jejuni* and are believed to have evolved from ε-proteobacteria and acquired by lateral-gene-transfer (Mogi et al., 2009). Low-potential quinones, such as menaquinone and rhodoquinone, are synthesized by microorganisms living under anaerobic or microaerophilic conditions, whilst ubiquinone is produced by microorganisms living under aerobic and microaerophilic environments (Unden et al., 2014; Nitzschke and Bettenbrock, 2018). Therefore, the ability of MQO from ε-proteobacteria to reduce ubiquinone in addition to menaquinone may have been advantageous for environmental adaptation by *Plasmodium* parasites, which have a complex life cycle that alternates between different hosts, oxygen requirements (e.g., aerobic in mammalian host, and microaerophilic in mosquito midgut), and are able to synthesize ubiquinone as well as menaquinone (Tonhosolo et al., 2010; Valenciano et al., 2019).

### 3.5 Insights into the reaction mechanism catalysed by *C. jejuni* MQO

Bi-substrate (A and B) enzymatic reactions, such as that of CjMQO, exhibit two main types of mechanisms: 1) sequential Bi-Bi and 2) ping pong Bi-Bi. The Lineweaver Burk plot representing 1/v *versus* 1/[A] at fixed [B], or 1/v *versus* 1/[B] at fixed [A], is characterized by intersecting or parallel lines in sequential (random or ordered) or ping pong mechanisms, respectively (Hartuti et al., 2018; Acharjee et al., 2021). The steady-state kinetic mechanism of purified CjMQO was investigated at different concentrations of UQ1 under a set of fixed malate concentrations (i.e., 0.5, 2, and 10 mM). The results showed lines intersecting at the left of 1/v axis indicating that CjMQO has an apparent bi-substrate, sequential Bi-Bi reaction mechanism (Figures 2C, D; Supplementary Figure S10) during which ternary complexes are formed (CjMQO/Malate/Quinone). The reaction mechanism observed for MQOs are unusual amongst other ETC dehydrogenases, such as dihydroorotate dehydrogenase and Type 2 NADH dehydrogenase, which show a ping-pong mechanism of catalysis (Takashima et al., 2002; Yano et al., 2014). Considering the α-value higher than 1 for the observed mixed-type inhibition of ferulenol and embelin *versus* malate, and the noncompetitive nature *versus* quinone, this would suggest the binding of malate occurs first followed by the binding of quinones, which is the opposite binding sequence to what was observed for mitochondrial MQOs (Hartuti et al., 2018; Acharjee et al., 2021). In such case, the two electrons from malate are first transferred to the FAD, producing FADH_2_, and then to the quinone, with bound FAD functioning as the redox centre. This is consistent with the spectral changes observed for CjMQO as the bound FAD was completely reduced by malate in the absence of quinone (Supplementary Figure S2).

### 3.6 Inhibition of *C. jejuni* growth by MQO inhibitors

To investigate the potential of CjMQO as a candidate drug target and its importance for *C. jejuni* survival, we examined the effect of ferulenol, a mitochondrial MQO inhibitor previously reported, and we show for the first time that embelin is also a potent inhibitor of CjMQO. Ferulenol, a sesquiterpene prenylated coumarin derivative, is found at high concentrations in *Ferula communis* (also known as Giant fennel), a plant that has numerous biological applications (Akaberi et al., 2015) with anti-coagulant (Monti et al., 2007), anticancer (Lariche et al., 2017), and antimicrobial (Appendino et al., 2004) activities. Furthermore, ferulenol is a promiscuous inhibitor with several targets (Acharjee et al., 2021). Embelin (2,5-dihydroxy-3-undecyl-p-benzoquinone) is a redox active benzoquinone (Joshi et al., 2009) that occurs naturally in *Embelia ribes* Burm. f.; a medicinal plant commonly known as Vidang in Sanskrit (India) and is widely used to treat a variety of diseases (Li et al., 2019). Moreover, embelin is a partial uncoupler of the ETC and may inhibit oxidative phosphorylation (Makawiti et al., 1990). Although embelin has never been described as an inhibitor of ETC dehydrogenase, our routine screening found that embelin is a mild inhibitor of *P. falciparum* MQO (IC_50_ of 0.203 μM; unpublished). Thus, embelin was tested against CjMQO in this study.

**FIGURE 3:**
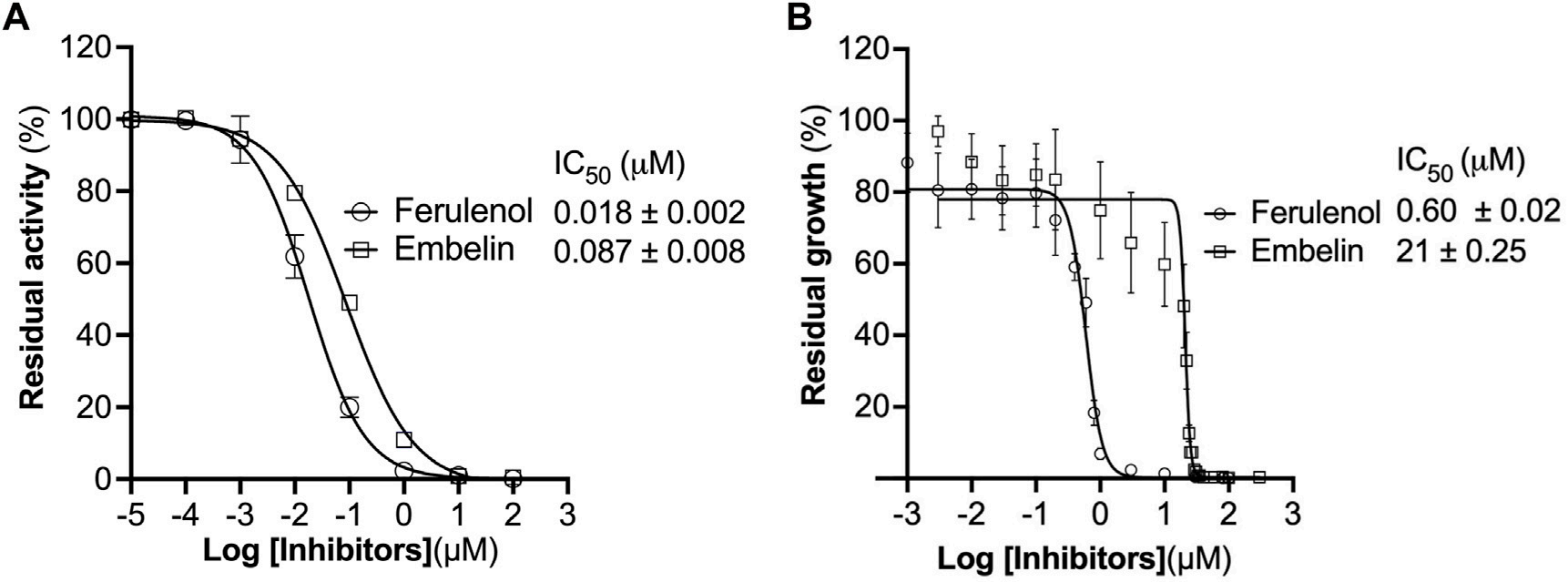
Effect of ferulenol and embelin on CjMQO and *C. jejuni* growth. **(A)** Inhibition of purified CjMQO by ferulenol and embelin. The residual enzymatic activity was measured in a mixture containing 50 mM MOPS pH 7.0, 100 μM UQ1, 1 mM KCN, 0.2 μg/mL of purified CjMQO, and varying concentrations of embelin and ferulenol. The reaction was started by the addition of 10 mM malate. Values represent the average of triplicate measurements. **(B)** *In vitro* effect of embelin and ferulenol on the growth of *C. jejuni* ATCC29428. *Campylobacter jejuni* was cultured in Muller Hinton broth for 24 h with a starting OD_600_ of 0.01 in the presence of varying concentrations of ferulenol and embelin. Kanamycin and ciprofloxacin (50 μg/mL each) were used as the positive control (100% inhibition). The resazurin assay was used to determine the residual growth on 96-well plates. Values represent the average of triplicate values.

We checked the effect of ferulenol and embelin on purified CjMQO and the results showed that these two compounds potently inhibited CjMQO, with IC_50_s of 0.018 and 0.087 μM, respectively (Figure 3A). The IC_50_ of ferulenol for CjMQO was three- and 46-times lower compared to that of PfMQO (0.057 μM) and TgMQO (0.822 μM), respectively. The inhibition constant (*K*_i_) of ferulenol was 0.02 μM and 0.01 μM *versus* UQ1 and malate, and of embelin was 0.14 μM and 0.10 μM *versus* UQ1 and malate, respectively. Using kanamycin and ciprofloxacin as positive controls, we tested the effect of ferulenol and embelin on the culture of *C. jejuni* ATCC29428. The results revealed that the growth of this bacterial strain was inhibited by both ferulenol and embelin displaying IC_50_s of 0.60 and 21 μM, respectively (Figure 3B). It has been reported that ferulenol displays cytotoxicity for DLD-1 (colon cancer-derived), Panc-1 (pancreatic cancer-derived), and human dermal fibroblast at IC_50_s of 50.1, 50.2, and 65.8 μM (Hartuti et al., 2018), respectively, corresponding to selectivity ranging from 83.5 to 110 towards *C. jejuni*. At a concentration of 100 μM, embelin completely inhibited the growth of *C. jejuni* ATCC29428. This concentration is still below the toxic concentration reported for human fibroblasts (IC_50_ of 739 μM) and the minimal inhibitory concentration (∼170–340 μM) reported for *E. coli*, methicillin-resistant *Staphylococcus aureus*, dermatophytic fungi, *Epidermophyton floccosum*, *Microsporum canis, Trichophyton mentagrophytes* and other pathogens (Feresin et al., 2003). As described above, ferulenol and embelin exert a variety of effects on cells; their ability to inhibit the growth of *C. jejuni* ATCC29428, as observed in this study, likely involves complex mechanisms that are not restricted to MQO targets, and should not be used in humans. Therefore, further studies are necessary to identify more specific MQO inhibitors.

### 3.7 Mechanism of *C. jejuni* MQO inhibition by ferulenol and embelin

**FIGURE 4:**
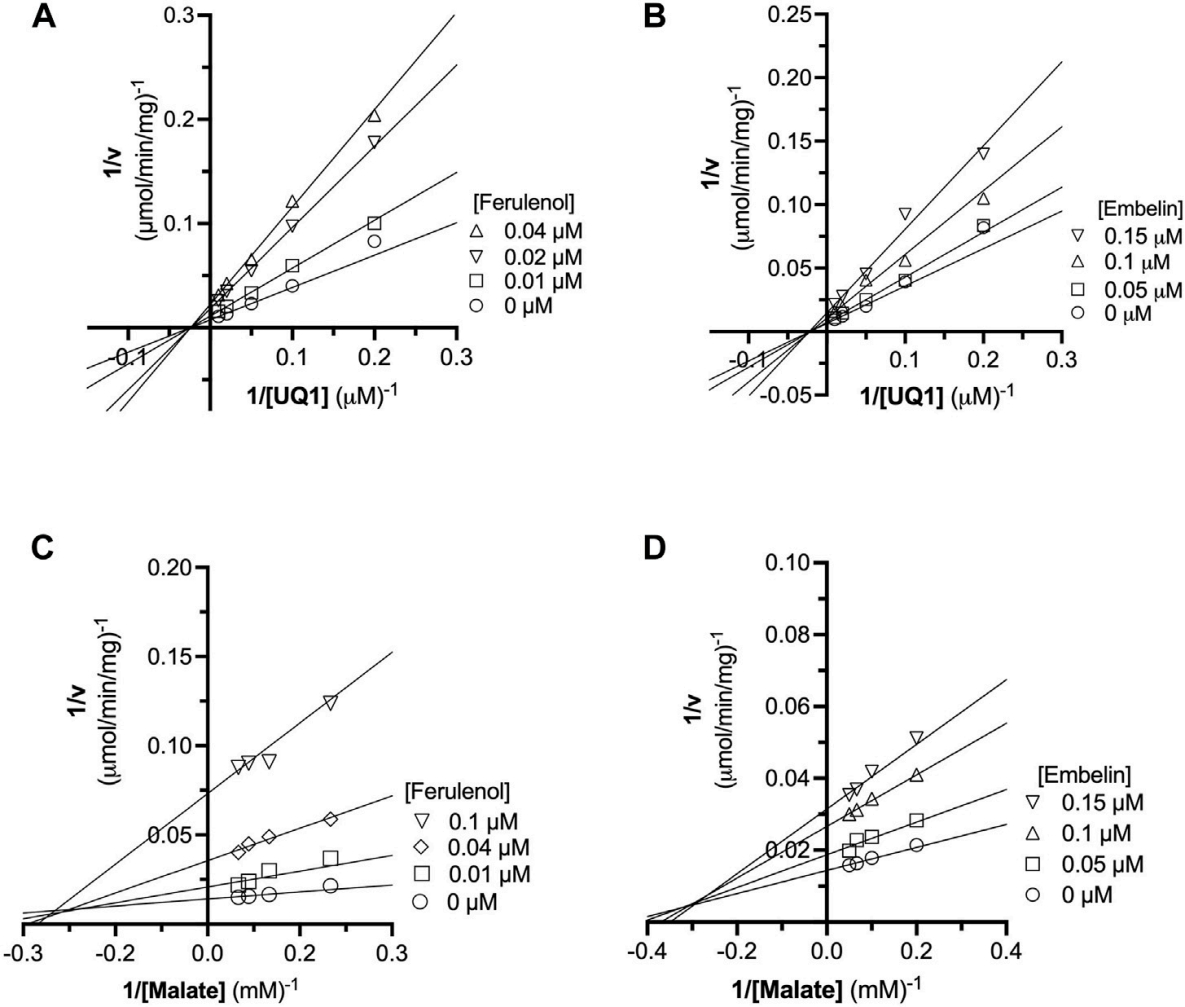
Analysis of ferulenol and embelin inhibition mechanism using double reciprocal plot. The inhibition mechanism of ferulenol was determined by varying the concentrations of UQ1 **(A)** or malate **(C)** while maintaining the concentrations of ferulenol. Similarly, the inhibition mechanism of embelin was performed by changing the concentration of UQ1 **(B)** or malate **(D)** at fixed concentrations of embelin. In all scenarios, the double reciprocal plot yielded straight lines intersecting in the second quadrant or at X-axis. Further analyses by fitting the data to the general mixed-type equation (Table 3) and using graphical methods (Supplementary Figures 12, 13), further supports that embelin and ferulenol were noncompetitive inhibitors *versus* quinone and mixed-type *versus* malate.

The inhibition mechanisms of the two inhibitors were determined by checking the residual enzymatic activity of CjMQO at different concentrations of UQ1 (Figures 4A, B) or malate (Figures 4C, D) under a set of fixed ferulenol or embelin concentrations. All of the double reciprocal plots obtained show intersecting lines, either in the second quadrant or on the X-axis (Figure 4). However, further analysis by fitting the data to a general equation of mixed-model inhibition using a nonlinear method (*R* ^2^ = 0.999), confirmed that the inhibition mechanism was mixed-type for both inhibitors *versus* malate with α values of 2.66 and 1.43 for ferulenol and embelin, respectively. Conversely, the inhibition mechanism was noncompetitive for both compounds *versus* quinones with α values of 1.04 and 0.998 for ferulenol and embelin, respectively (Table 3). These findings corroborated those obtained by the graphical method with Dixon and Cornish Bowden plots (Supplementary Figures 11, 12). These α values suggest that the two inhibitors had the potential to bind to both free CjMQO as well as CjMQO/substrates complex with slightly high preference for free CjMQO in case of inhibitor *versus* malate. Although this mechanism is consistent with previously reported inhibition mechanisms for ferulenol on TgMQO (Acharjee et al., 2021), it contrasts with the inhibition mechanism reported for PfMQO (Hartuti et al., 2018). The noncompetitive and mixed-type of inhibition suggest that 1) the concentration of the substrate does not influence the binding capacity of the two inhibitors, and 2) they can bind to both free enzymes (CjMQO) and enzyme-substrate complexes (Ring et al., 2014). Altogether, our biochemical analyses show that embelin and ferulenol bind to sites other than the malate and quinone binding sites.

**TABLE 3:** Kinetic parameters of the inhibition by ferulenol and embelin.

Inhibitor	Substrate		*V*_max_	*K*_m_	*K*_i_	*α*	Inhibition
	Varied	Fixed	(μmol/min/mg)	(μM)	(μM)		
Ferulenol	Malate	UQ1	70 ± 2	1,845 ± 201	0.01	2.66	Mixed
Ferulenol	UQ1	Malate	133 ± 7	42 ± 5	0.02	1.04	NC
Embelin	Malate	UQ1	71 ± 3	2,225 ± 292	0.10	1.43	Mixed
Embelin	UQ1	Malate	164 ± 11	47 ± 6.3	0.14	0.998	NC

The enzymatic activity of CjMQO *versus* inhibitor was measured as described in the materials methods section. The CjMQO kinetics data for the inhibition by ferulenol and embelin were fitted to a mixed-model equation in Graphpad Prism software. *V*_max_ and *K*_m_ represent the apparent maximal velocity and Michaelis constant, respectively. *K*_i_ is the inhibition constant while α determines the degree to which inhibitor affects the affinity of the enzyme for substrate. NC: noncompetitive type of inhibition.

## 4 Conclusion

In conclusion, our results support those of previous studies which showed a low recovery rate of transposon mutants of MDH and also MQO in *C. jejuni* (Gao et al., 2017), indicating that CjMQO is likely essential for *C. jejuni* survival and a potential drug target. Since the crystal structure of MQO is not yet known, the binding sites of ferulenol and embelin as well as the residues interacting with them remain to be clarified and studies on the crystallization of CjMQO are currently ongoing. Because *C. jejuni* also conserves the NAD^+^-dependent MDH (CjMDH), further studies, including the knockdown or knockout of the CjMQO/CjMDH genes, are necessary in order to elucidate the role of these enzymes in the pathophysiology of *C. jejuni* and to fully validate CjMQO as a novel drug target.

## Data Availability

The datasets presented in this study can be found in online repositories. The names of the repository/repositories and accession number(s) can be found in the article/Supplementary Material.
