# Supplementary material for: Biochemical characterization and identification of ferulenol and embelin as potent inhibitors of malate:quinone oxidoreductase from Campylobacter jejuni
Source: Front Mol Biosci. 2023 Jan 26;10:1095026. doi: 10.3389/fmolb.2023.1095026 (PMC9908594; doi:10.3389/fmolb.2023.1095026)
Supplement: Supplementary file 1 [file DataSheet1.DOCX]

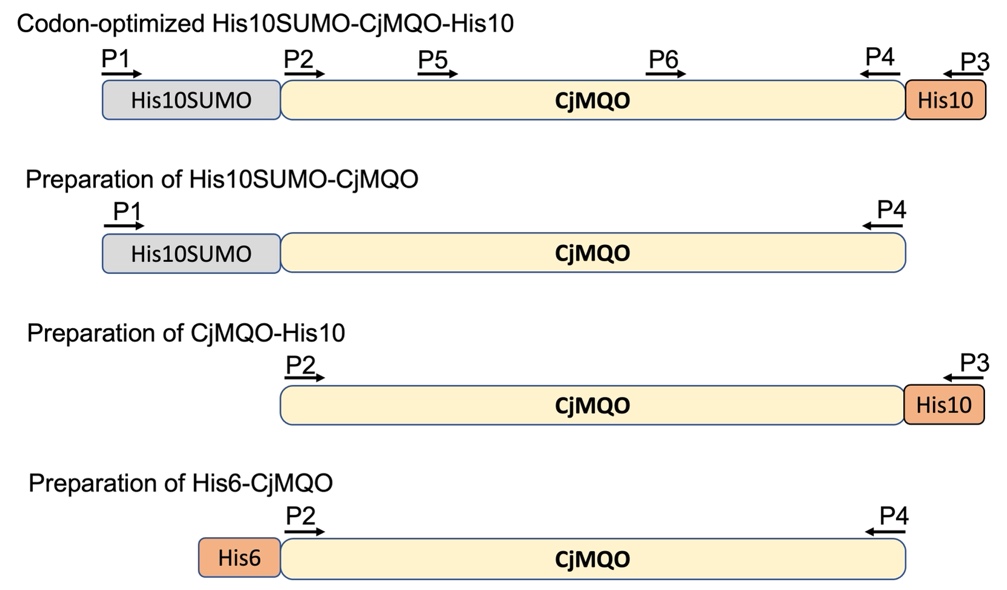


**Supplementary Figure 1**. Primers used to amplify the three CjMQO gene constructs. P1 corresponds to the forward primer for His10SUMO-CjMQO, P2 to the forward primer for CjMQO-His10 and His6-CjMQO, P3 to the reverse primer for CjMQO-His10, and P4 to the reverse primer for His10SUMO-CjMQO and His6-CjMQO. The primers P5 and P6 are similar for all constructs and were used for the colony PCR in combination with T7 forward or reverse primers. His10SUMO-CjMQO and CjMQO-His10 were cloned into pET101 and His6-CjMQO into pET151. The His6 tag from His6-CjMQO is from the pET151.


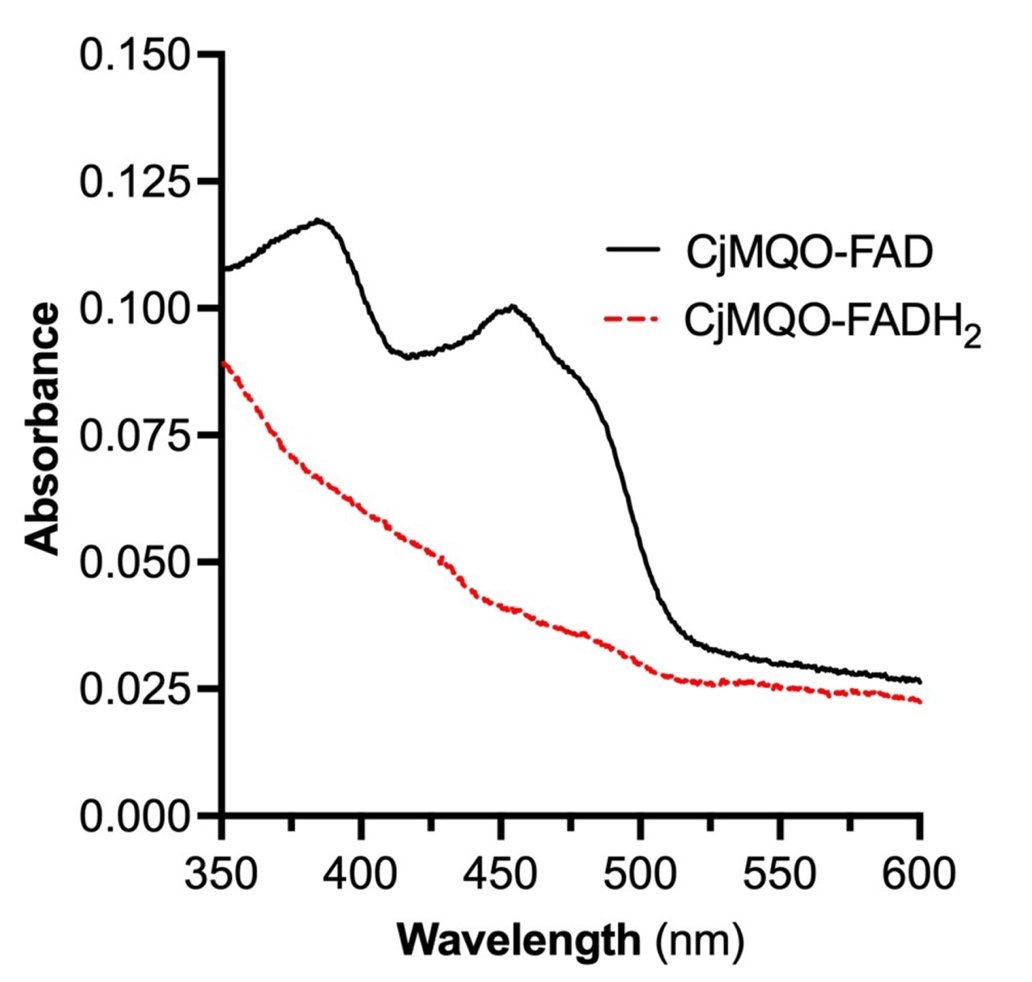


**Supplementary Figure 2.** UV-visible spectra of FAD bound to CjMQO in its oxidized (CjMQO-FAD) and reduced (CjMQO-FADH_2_) forms**.** After recording the background with MOPS buffer (pH 7.0), 8.5 μM of the CjMQO purified (in absence of FAD) was added and the spectrum of bound FAD was recorded (black line). The results show the peak of bound FAD at 450 nm. Next, malate was added to a final concentration of 1 mM and the spectrum was recorded again showing the disappearance of the peak at 450 nm (dashed red line), indicating that FAD was reduced (FADH_2_). Considering the extinction coefficient of 11.1 mM^-1^cm^-1^ at 450 nm, the absorbance at 450 nm (0.0993), suggests the presence 8.9 μM of FAD in the mixture which corresponds to the ratio of FAD:CjMQO of 1.05. But results from 3 independent experiments using different preparations of CjMQO show that the ratio FAD:CjMQO was in the range of 0.93 to 1.16 indicating that the actual ratio is 1.


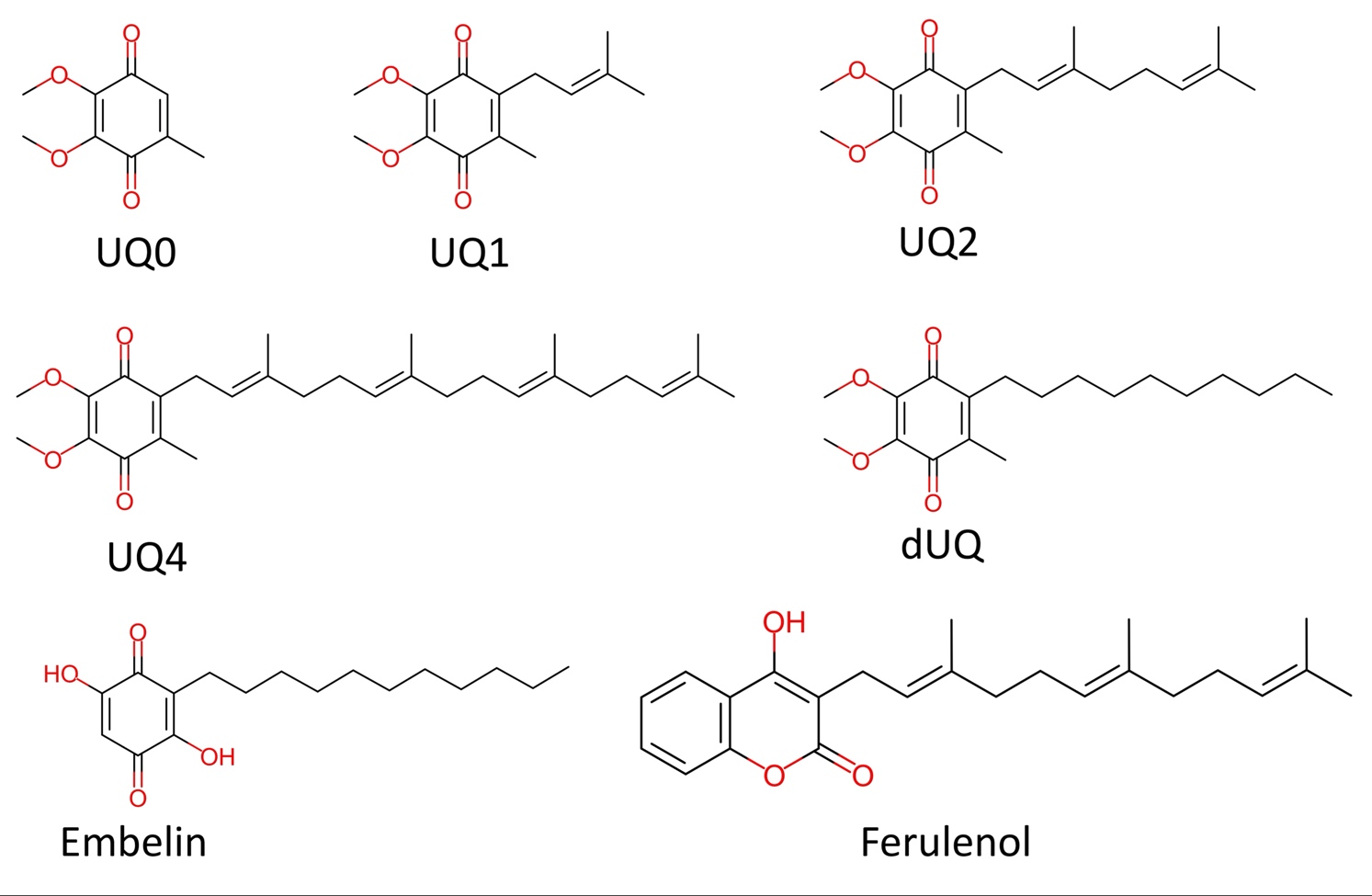


**Supplementary Figure 3.** Chemical structures of ubiquinones (UQ0, UQ1, UQ2, UQ4, and dUQ), embelin and ferulenol


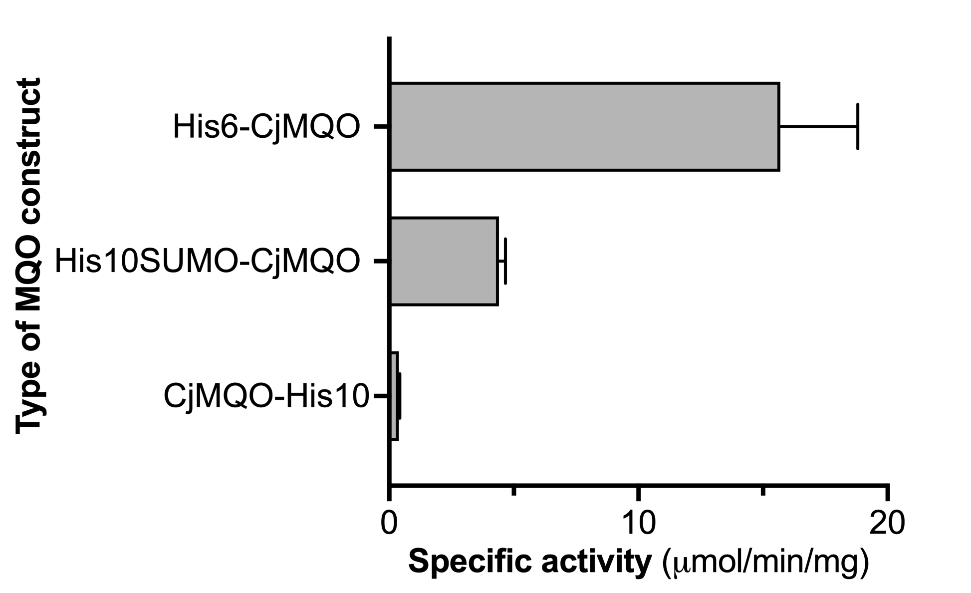


**Supplementary Figure 4.** Expression the three MQO constructs in *Escherichia coli*. The expression of CjMQO in the three constructs was induced separately with 20 µM IPTG in BL21 Star™ (DE3) and the membrane fractions were prepared following the protocol described in the Materials and Methods. The activity of membrane-bound CjMQO was assayed by DCIP reduction at 600 nm. Values on the graph represent the average of assays conducted in triplicate. The pET151/His6-CjMQO construct yielded the highest specific activity in the membrane fraction.

(**A**) (**B**)


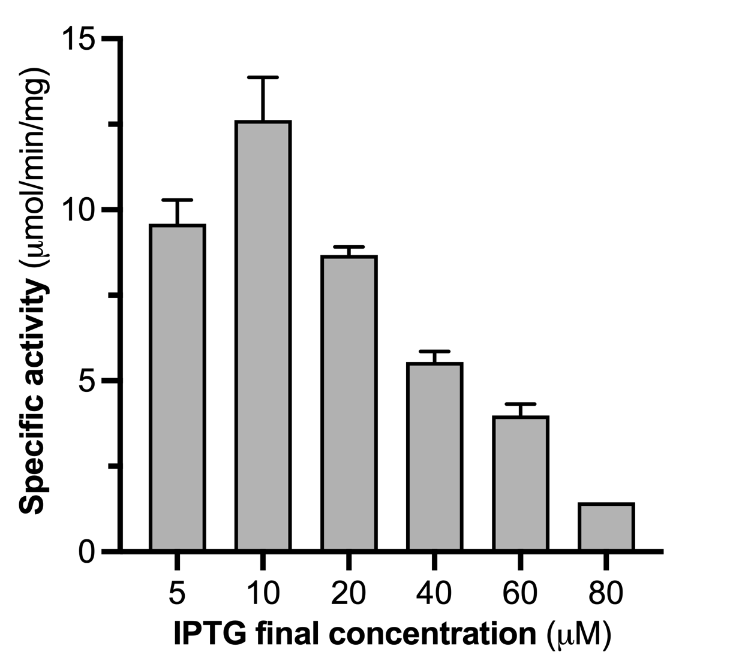

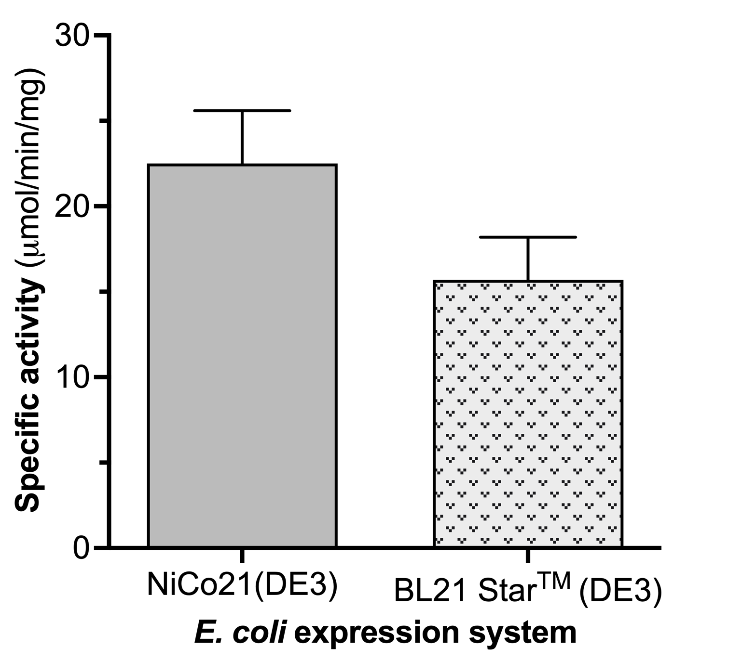


**Supplementary Figure 5.** Optimization of conditions for CjMQO expression. (A) Optimization of CjMQO induction by IPTG. BL21 Star™ (DE3) cells transformed with the pET151/His6-CjMQO were cultured as described in Materials and Methods, but for only 24 hours, and the expression was induced with varying concentrations of IPTG (5–80 µM). The results show that His6-CjMQO was strongly induced at low IPTG concentrations, with 10 µM yielding the highest enzymatic activity. (B) Expression of His6-CjMQO in NiCo21 (DE3) and BL21 Star™ (DE3). The comparison of the specific activity of His6-CjMQO in the membranes of both expression systems, induced by 10 µM of IPTG, shows that NiCo21 (DE3) yielded the highest specific activity in the membrane fraction. The values in the figures represent the average of DCIP assays (600 nm) conducted at 37°C in (A) duplicate and (B) from six independent experiments for BL21 Star™ (DE3) and two independent experiments for NiCo21 (DE3) where each data point was assayed in triplicate.

**
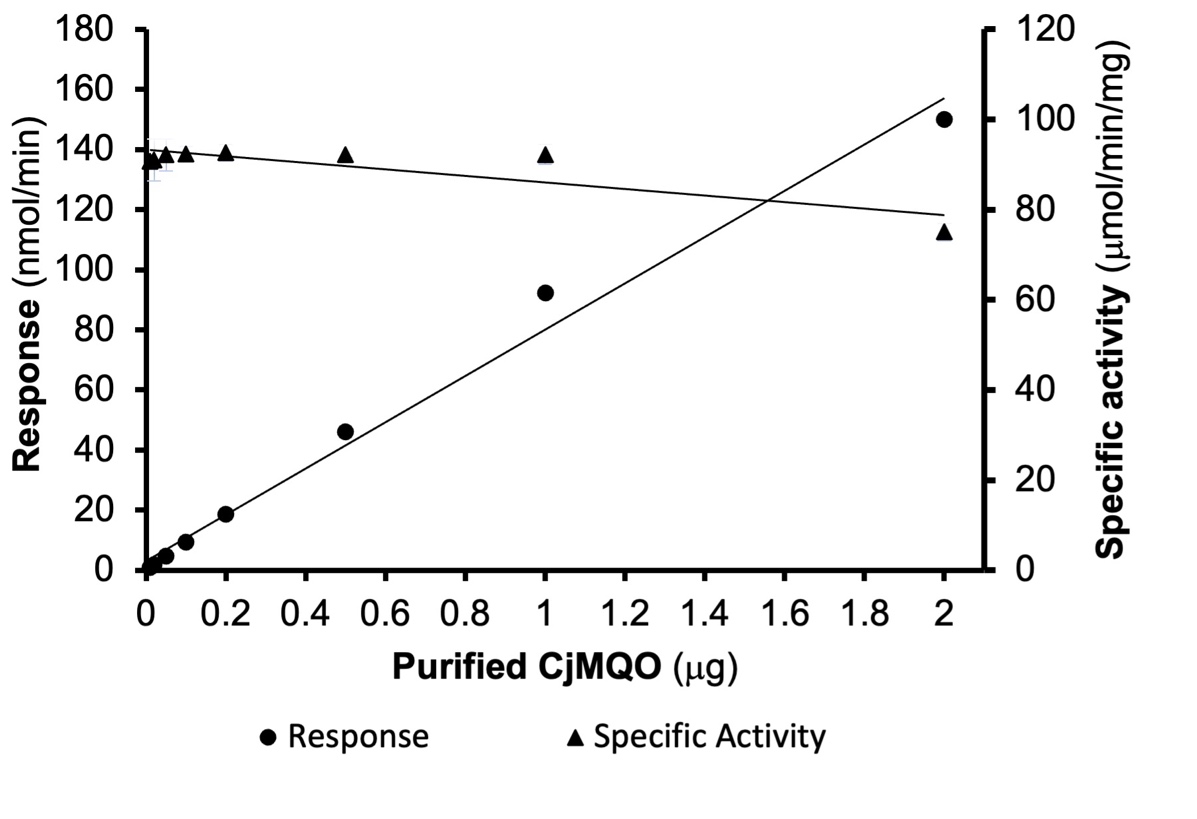
**

**Supplementary Figure 6.** Dose-response curve of purified CjMQO. The CjMQO activity was assayed spectrophotometrically using varying concentrations of purified CjMQO, i.e., 0.01–2 μg/mL in 1 mL reaction mixture containing 50 mM HEPES pH 7.0, 20 μM dUQ, and 120 μM DCIP. The reaction was started by adding 10 mM of malate to the reaction mixture and the reduction of DCIP was measured at 600 nm. The right and left axes represent the specific activity (μmol/min/mg) and the response (nmol/min), respectively. Values in this figure represent the average of assays that were conducted in triplicate at 37°C. Error bars, which are smaller than the symbol size, are automatically removed by GraphPad and not displayed.


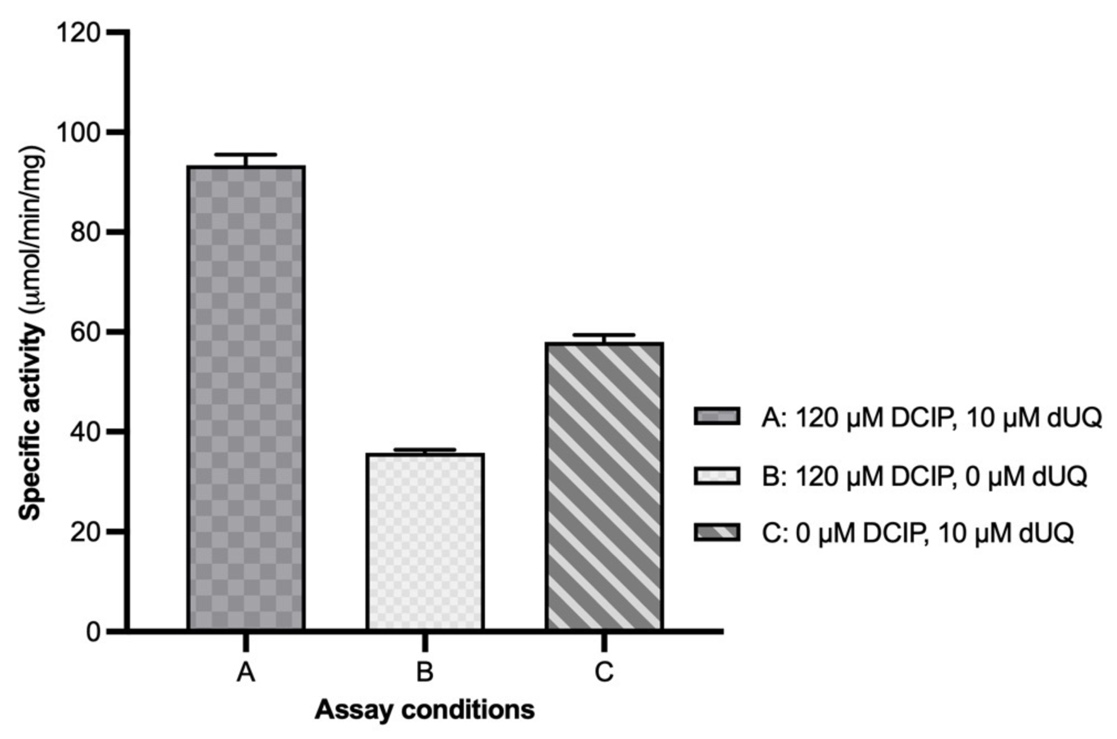


**Supplementary Figure 7**. Comparison of DCIP and UQ assays for CjMQO activity determination. CjMQO activity was determined based on the presence of DCIP and dUQ (A), DCIP only (B), and ubiquinone only (C) at 600 nm (A and B) and 278 nm (C), respectively. The reduction of DCIP was observed in the absence of dUQ (B), yielding almost one-third of the activity recorded in presence of both DCIP and dUQ. Values in this figure represent the average of assays conducted in triplicate at 37°C.

(A) (B)


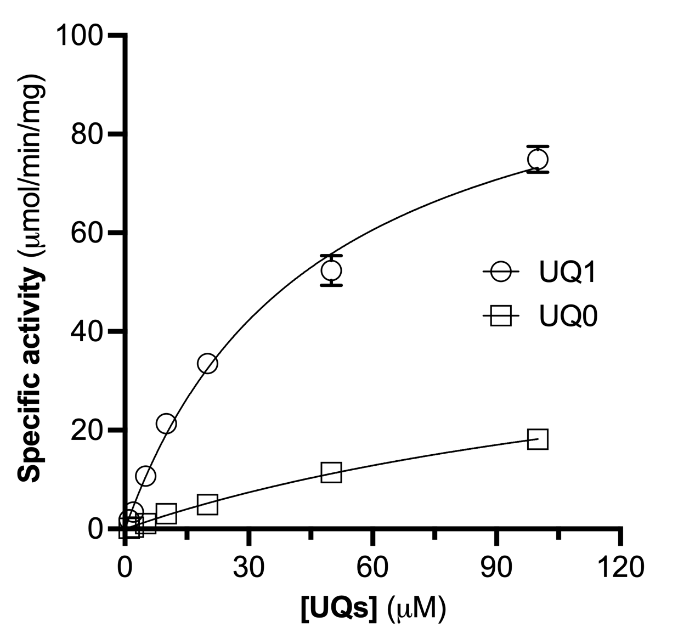

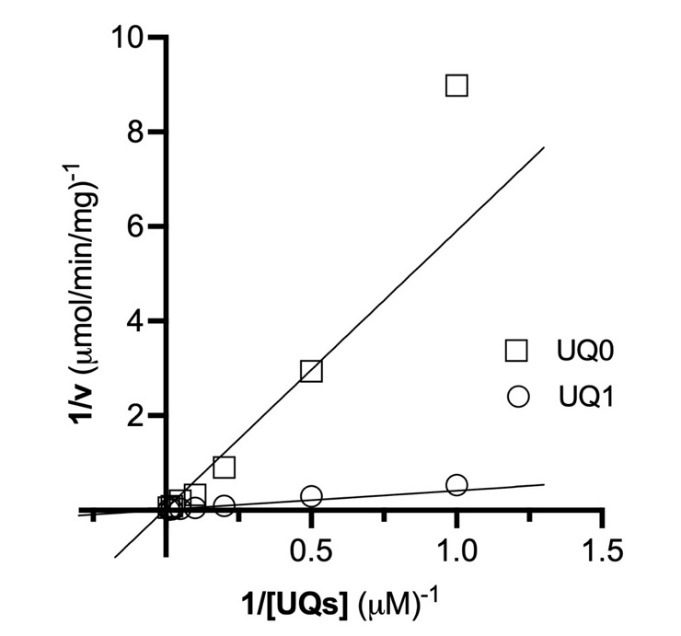


(C) (D)


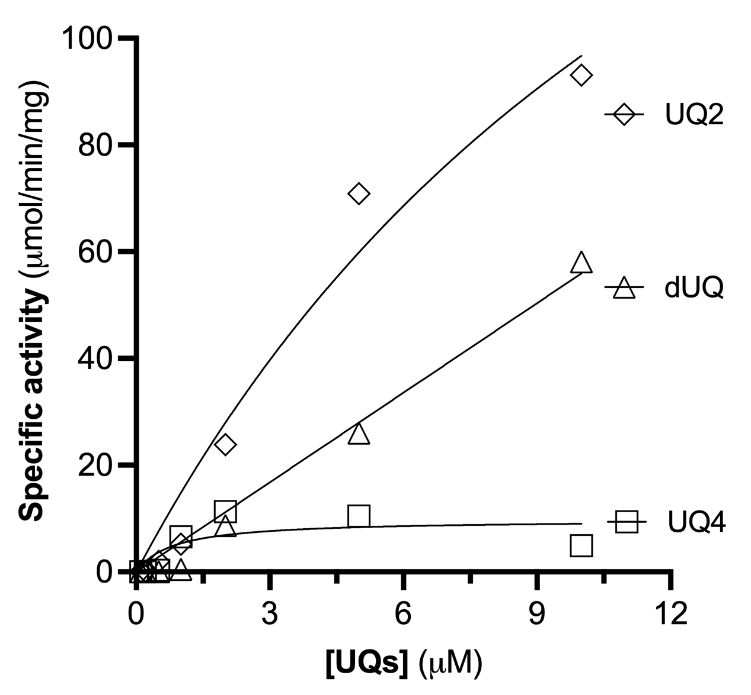

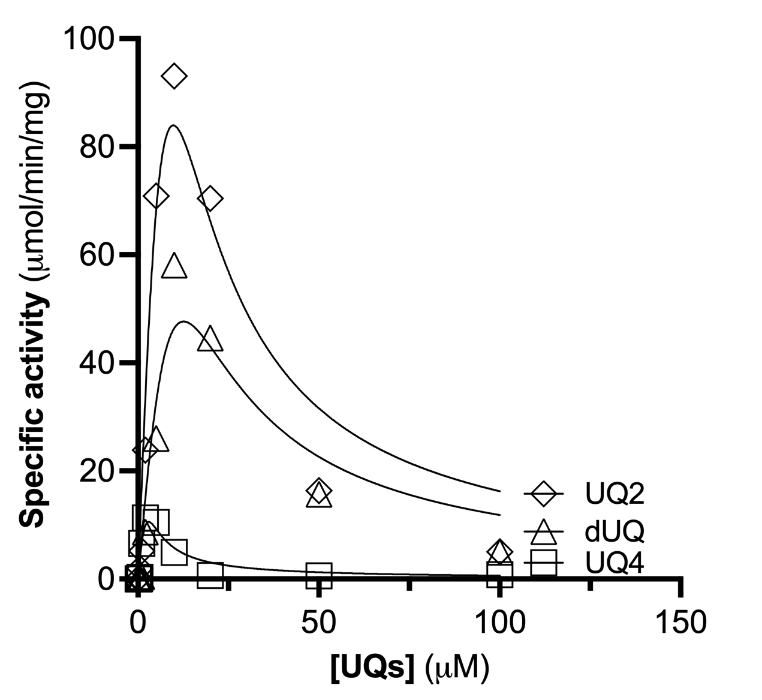


**Supplementary Figure 8**. Kinetics of purified CjMQO in the presence of different ubiquinone concentrations. (A) Michaelis-Menten and (B) Lineweaver-Burk plots for UQ0 and UQ1. (C) Michaelis-Menten plot for UQ2, UQ4 and dUQ at a lower concentration range, and (D) substrate inhibition of CjMQO activity by UQ2, UQ4 and dUQ. Values in these figures represent the average of assays conducted in triplicate at 37°C. Values of initial velocity that are lower than expected on the hyperbolic line at low concentrations of the quinones might be due to the consumption of quinones during the time delay between mixing the substrate and recording. At high concentrations of long side chain quinones (D), the sharp decrease in the specific activity might be due to the low solubility of these quinone species in our assay condition. Error bars which are smaller than the symbol size are automatically removed by GraphPad and not displayed.

(**A**) (**B**)


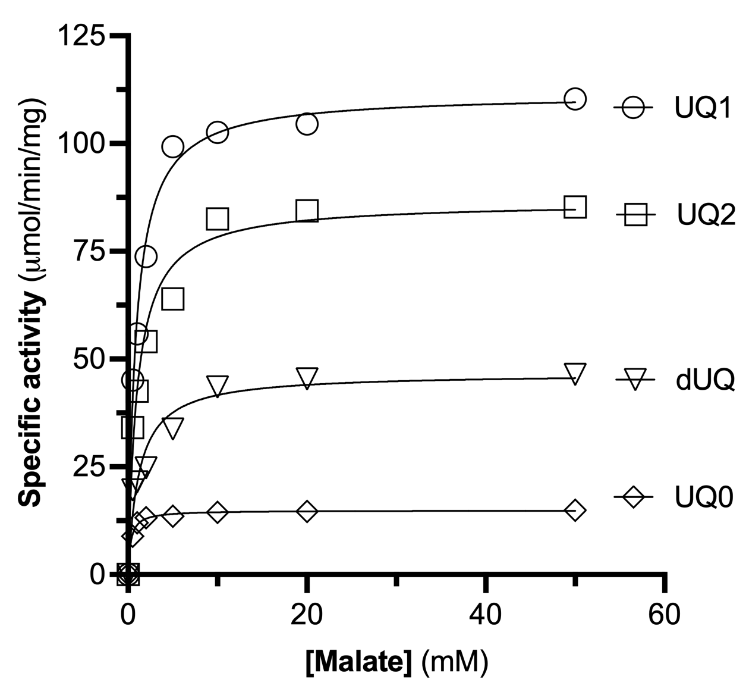

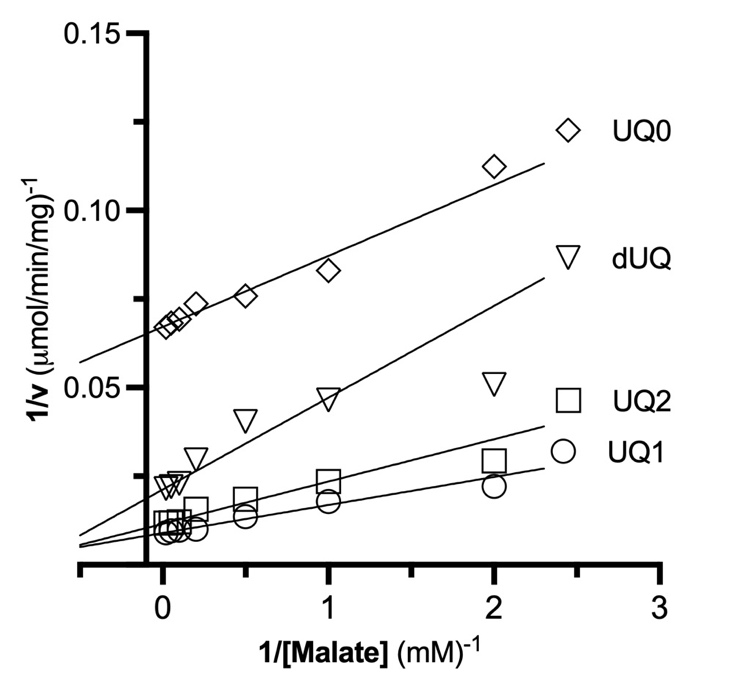


**Supplementary Figure 9**. Kinetics of CjMQO in the presence of varying malate concentrations. (A) Michaelis-Menten and (B) Lineweaver-Burk plots for UQ0, UQ1, UQ2, and dUQ. Values in these figures represent the average of assays conducted in triplicate at 37°C. Error bars, which are smaller than the symbol size, are automatically removed by GraphPad and not displayed.


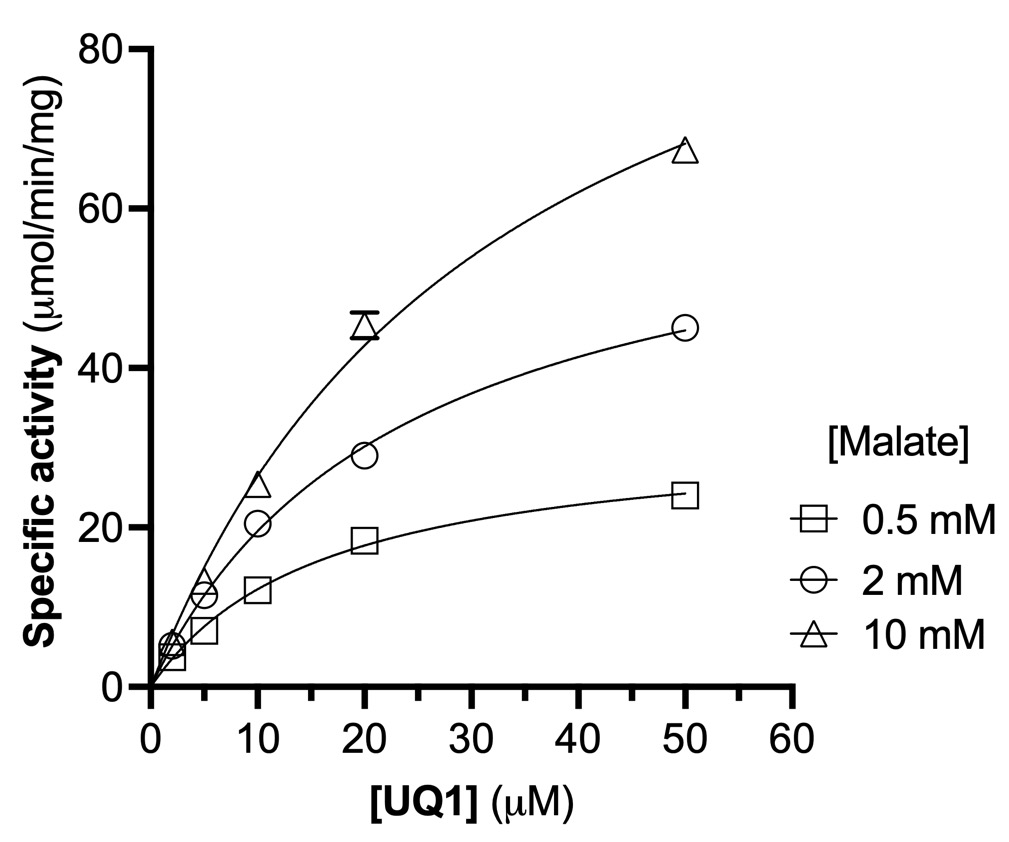


**Supplementary Figure 10.** Michalis-Menten plot for the analysis of the kinetic mechanism of CjMQO. The activity of purified CjMQO was measured at varying UQ1 concentrations at fixed malate concentrations (i.e., 0.5, 2, and 10 mM). The *K_m_* values were 16.3, 23.8 and 32.6 μM and *V_max_* were 32.1, 66.0 and 112.6 μmol/min/mg for fixed the concentration of malate fixed at 0.5, 2 and 10 mM, respectively. The Lineweaver-Burk plot is shown in the Figure 2C. Error bars, which are smaller than the symbol size, are automatically removed by GraphPad and not displayed.


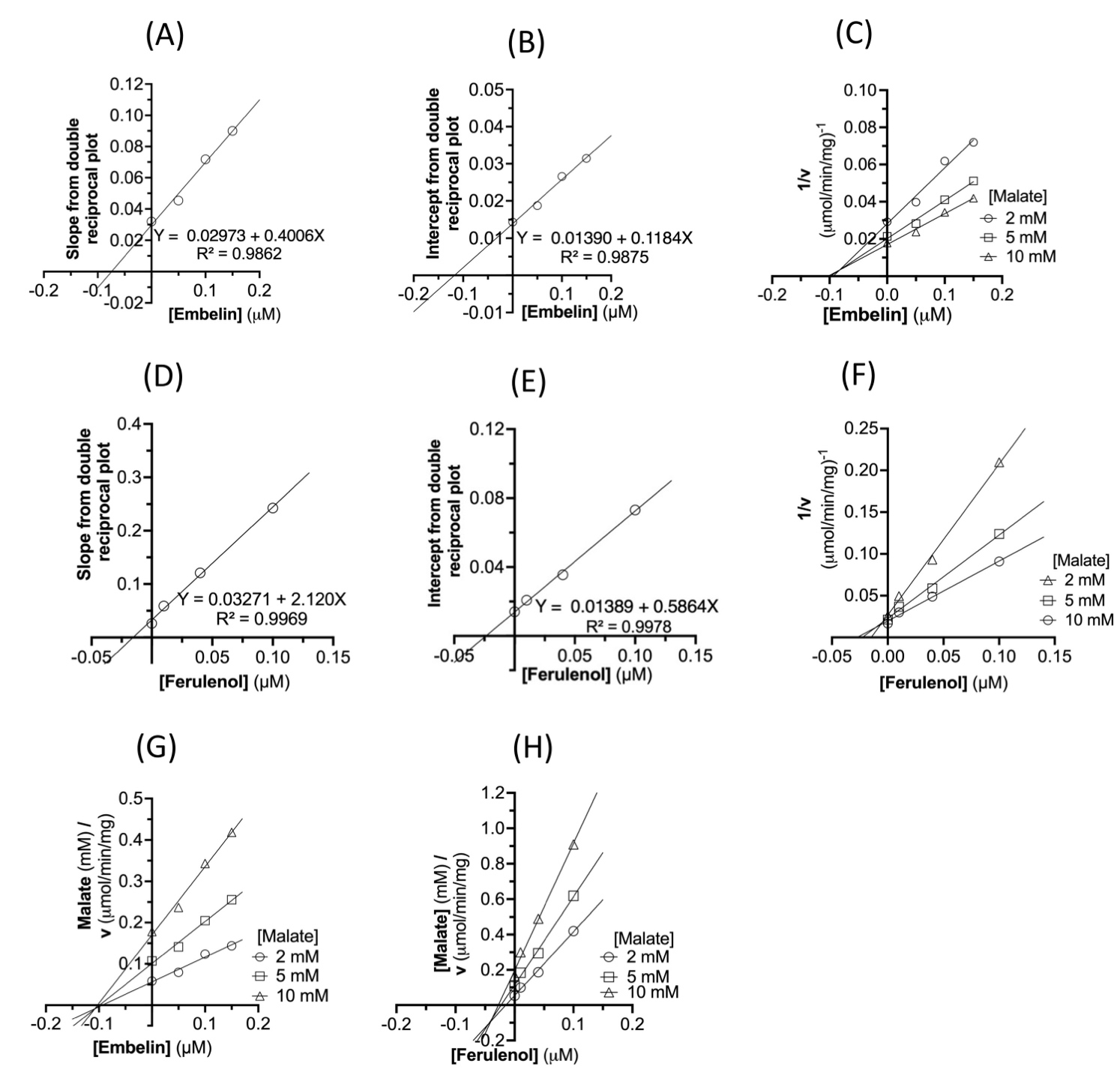


**Supplementary Figure 11. Analysis of the inhibition mechanism of embelin and ferulenol *versus* malate.** The inhibition mechanism was assayed as described in Materials and Methods section and the Lineweaver-Burk plots are presented in figure 4C &D**.** Linear replots of slope (A,D) and intercept (B, E) as function of inhibitor are shown. As the pattern of Dixon plots (C, F) did not distinguish between competitive and mixed-type inhibition, Cornish-Bowden diagram (G, H) were plotted and showed results consistent with mixed-type inhibition.


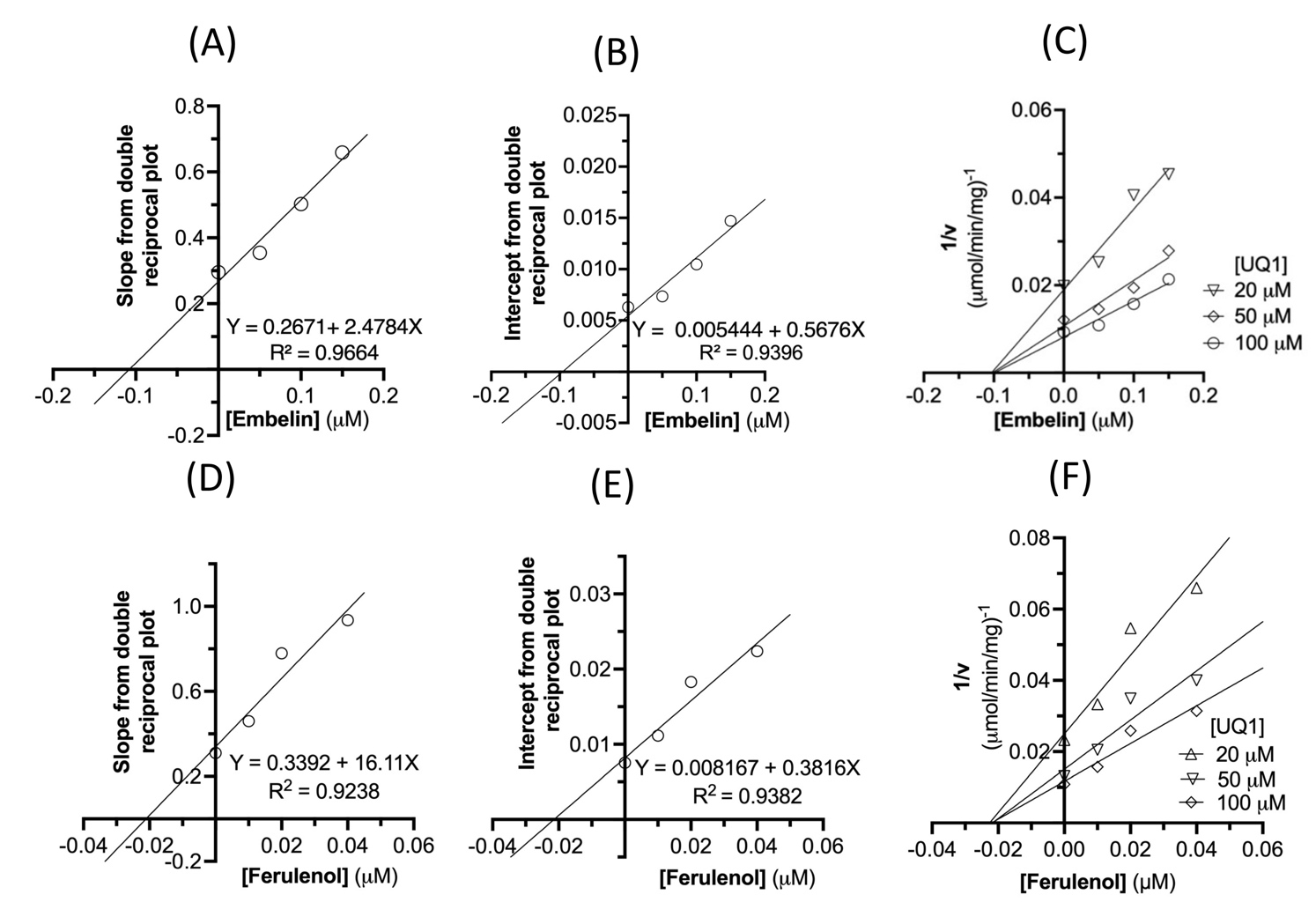


**Supplementary Figure 12. Analysis of the inhibition mechanism of embelin and ferulenol *versus* UQ1.** The inhibition mechanism was assayed as described in Materials and Methods section and the Lineweaver-Burk plots are presented in figure 4A &B**.** Replots of slope (A,D) and intercept (B, E) as function of inhibitor were poorly linear. The inhibition mechanism was analysed with Dixon plot which shows lines meeting at the X-axis, indicating a noncompetitive mechanism of inhibition.
